# Development of silicon-fluorescein-based photolabile protecting groups with enhanced uncaging quantum yield

**DOI:** 10.1039/d5ra09470d

**Published:** 2026-03-02

**Authors:** Naoya Ieda, Miyu Tachi, Misuzu Noda, Mei Harada, Yuji Hotta, Kazuki Kondo, Haruka Tsuchiya, Mikako Ogawa, Mitsuyasu Kawaguchi, Hidehiko Nakagawa

**Affiliations:** a Graduate School of Pharmaceutical Sciences, Hokkaido University N12 W6, Kita-ku Sapporo Hokkaido 060-0812 Japan ieda@pharm.hokudai.ac.jp; b Graduate School of Pharmaceutical Sciences, Nagoya City University 3-1, Tanabe-dori, Mizuho-ku Nagoya Aichi 467-8603 Japan deco@phar.nagoya-cu.ac.jp; c Graduate School of Medical Sciences, Nagoya City University 1, Kawasumi, Mizuho-cho Nagoya Aichi 467-8601 Japan; d School of Pharmaceutical Sciences, Hokkaido University N12 W6, Kita-ku Sapporo Hokkaido 060-0812 Japan; e WPI-ICReDD, Hokkaido University N21 W10, Kita-ku Sapporo Hokkaido 001-0021 Japan

## Abstract

Photolabile protecting groups (PPGs) that respond to visible light are valuable tools for spatiotemporal control of biological events. However, achieving high uncaging efficiency at biocompatible wavelengths remains a significant challenge. In this study, we report a new class of PPGs triggered by photoinduced electron transfer (PeT) and activated by orange light (*ca.* 600 nm), designed to overcome this limitation. Using a structure-based approach assisted by quantum-chemical calculations, we focused on minimizing the activation energy (Δ*E*_a_) of the bond cleavage step following PeT. By rationally tuning the picolinium cation and antenna moieties, we achieved significantly improved reaction quantum yields, surpassing conventional PeT-based systems operating in this window. These results were consistent with the predicted energetics of the post-PeT intermediates, validating our design strategy. The practical utility of the system was demonstrated through the design and synthesis of a photoactivatable prodrug of a vasodilator. Upon orange light irradiation, the compound induced vasodilation in a sustained and controllable manner. This work not only provides a new strategy for designing high-efficiency PPGs operating at biologically compatible wavelengths, but also highlights the importance of combining quantum-chemical predictions with molecular design. Furthermore, the generality of the approach suggests its applicability to other single-electron-driven reactions. We believe these findings open a new avenue for the rational development of visible-light-responsive molecular tools in chemical biology and photopharmacology.

## Abstract 2

Quantum chemistry-guided transition state stabilization of Si-fluorescein photolabile protecting groups achieved a 70-fold increase in uncaging efficiency, enabling the precise optomanipulation of biological events using orange light.

## Graphical abstract

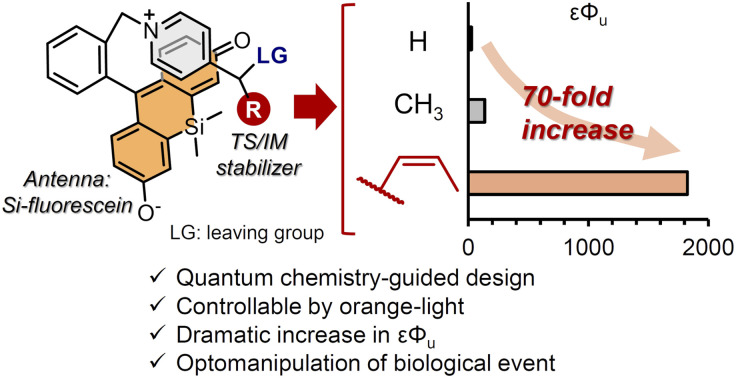

## Introduction

Caged compounds are bioactive molecules protected with a protecting group, which is cleaved and activated by a specific stimulus. Among various stimuli, light is particularly advantageous because it enables spatiotemporally precise activation, and thus photolabile protecting groups (PPGs) have been widely employed to regulate the activity of bioactive molecules.^1,2^ A wide variety of PPGs have been developed, serving not only as tools to dissect biological processes with high precision, but also as candidate therapeutic agents.^3–5^ However, the most commonly used PPGs, such as the 2-nitrobenzyl-type and coumarin-4-methyl-type PPGs, require harmful ultraviolet to purple light irradiation (300–400 nm) for the uncaging reaction.^1,6^ It would be preferable to use visible light in order to avoid photodamage to cells or tissues.^7^ Although some visible-light-driven PPGs have been reported, their development is still challenging because it is difficult to supply sufficient visible light energy to cleave chemical bonds.^8–16^

**Fig. 1:**
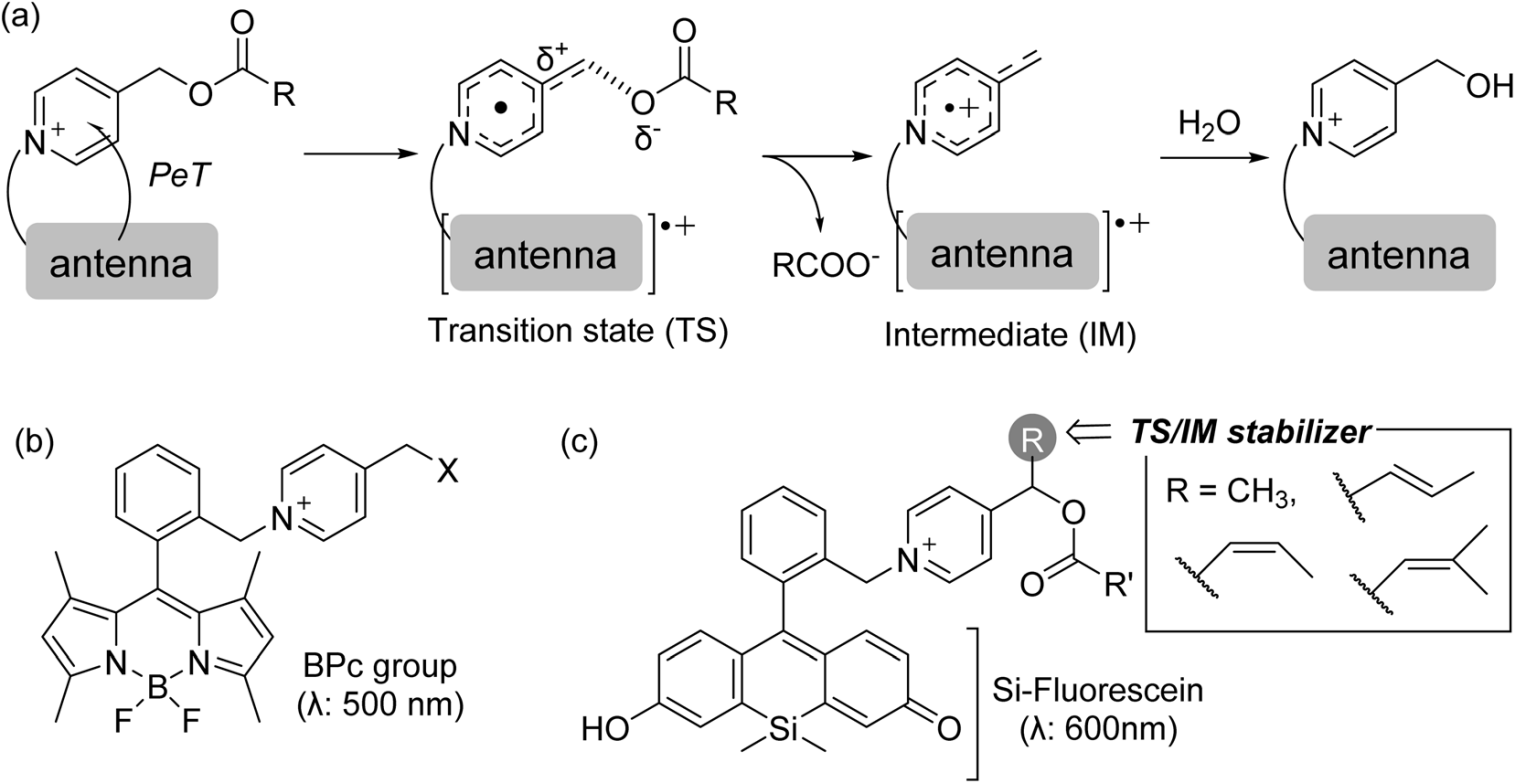
A plausible mechanism of PeT-driven picolinium cation photolysis (a); structure of BODIPY-picolinium conjugate as a blue-light-responsive PPG (b); design of orange-light-responsive PPGs bearing TS/IM stabilizers (c).

To achieve spatiotemporal control of the release of nitric oxide (NO), which exhibits a potent vasorelaxant effect, we have developed photoinduced-electron-transfer-driven NO releasers (PeT-driven NO releasers) which efficiently release NO in response to visible light irradiation up to a wavelength of 660 nm, making them applicable for controlling vasodilation even *in vivo*.^17–21^ To further expand the utility of this PeT-driven strategy to other bioactive compounds, we have focused on the *N*-alkyl-4-picolinium group as a candidate to undergo electron transfer from photoactivated dyes, followed by C–O bond scission.^22–25^ To test this approach, we previously synthesized a BODIPY-picolinium conjugate (BPc group) as a blue-light-responsive PPG capable of protecting and releasing both amine and carboxylic acid derivatives (Fig. 1a and b).^26^ However, blue light can be toxic because endogenous dyes, such as porphyrin, flavin, and melanin, can be excited to produce reactive oxygen species, and the BPc group also has the drawback of a low reaction quantum yield.^27–33^ Moreover, no predictive system based on computational chemistry has yet been established for estimating the reaction efficiency of caged compounds triggered by PeT. Such knowledge is expected to provide important insights for the rational design of functionalized caged compounds. In this work, we report novel PeT-triggered PPGs responsive to 600 nm light, which is longer than the excitation wavelength of conventional BPc-based systems (500 nm). Guided by quantum-chemical calculations, we designed structural modifications to the picolinium cation aimed at lowering the activation barrier of the bond cleavage step. The synthesized compounds exhibited significantly improved quantum yields, as predicted, and we confirmed their utility for the light-controlled release of a vasodilatory compound.

## Results

As an antenna moiety for PeT-triggered PPGs responsive to longer wavelengths than the BPc group, we focused on silicon-fluorescein (Si-fluorescein), a fluorescein derivative in which the 10-position is substituted with a dimethylsilyl group.^34,35^ Si-fluorescein possesses an acidic proton, and its deprotonated form exhibits strong absorption at around 600 nm (Fig. 1c). Given its long-wavelength absorption and anionic nature, we envisioned that Si-fluorescein would interact electrostatically with the positively charged picolinium cation. This ionic interaction and the favorable spectroscopic properties were expected to facilitate efficient photoinduced electron transfer, leading to a new class of PPGs activatable by orange light (around 600 nm). In our previous work, we reported a PeT-triggered PPG that employed BODIPY as the antenna. Comparison of the HOMO–LUMO energy levels of BODIPY and Si-fluorescein revealed that Si-fluorescein possesses higher-lying frontier orbitals (Fig. S1). This indicates that efficient electron transfer from Si-fluorescein to the picolinium cation is also feasible, supporting the suitability of Si-fluorescein as an antenna for PeT activation. To further improve the quantum yield of the photoreaction, we sought to lower the activation energy (Δ*E*_a_) of the bond-cleavage transition state following the PeT process. In addition, to stabilize the radical intermediate formed after bond dissociation, we introduced substituents at the sp^3^ carbon of the picolinium cation to stabilize either a *δ*^+^-carbon in the transition state (TS) or a radical cation intermediate (IM)—we refer to such substituents as “TS/IM stabilizers” (Fig. 1c).^36,37^ To achieve electronic stabilization through hyperconjugation and extended π-conjugation, we selected methyl, 2-methylvinyl, and 2,2-dimethylvinyl groups as representative TS/IM stabilizers. To predict how the TS/IM stabilizers affect the bond-cleavage reaction, we performed quantum-chemical calculations to evaluate the transition state energies and the free energy changes before and after C–O bond cleavage in the one-electron-reduced state of the picolinium cation. To simplify the calculations, we did not use the full structures as shown in Fig. 1c, but instead omitted the Si-fluorescein structure and performed calculations on the one-electron-reduced picolinium cation derivatives S1–S5, in which a model acetic acid moiety is attached to mimic the protected structure (Fig. S2). While full-scale calculations including the chromophore would provide detailed insights from PeT to cleavage, the computational demands are substantial. Consequently, we utilized a simplified model in this study to selectively analyze the bond cleavage reactivity.

The calculations were performed using the UωB97X-D functional with the cc-pVDZ basis set and the PCM solvation model (water, see SI).^38,39^ The computational results showed that both Δ*E*_a_ required to reach the transition state and the Gibbs free energy change (Δ*G*) decreased or became more negative in the order of S2 < S4 < S5 < S3. In the case of S1, a relaxed scan along the C–O bond distance revealed no clear downhill pathway toward dissociation according to this computational method, making it difficult to obtain reliable values. For the other compounds, all of the calculated Δ*E*_a_ values were below 10 kcal mol^−1^, suggesting that these reactions are feasible under ambient conditions. These findings suggest that, in the bond-cleavage process following PeT, stabilization of the radical cation through hyperconjugation and extension of the π-conjugated system—enabled by appropriate sp^2^ carbon substituents—effectively lowers Δ*E*_a_ and shifts Δ*G* to more negative values, thereby significantly enhancing the efficiency of the cleavage reaction.

**Fig. 2:**
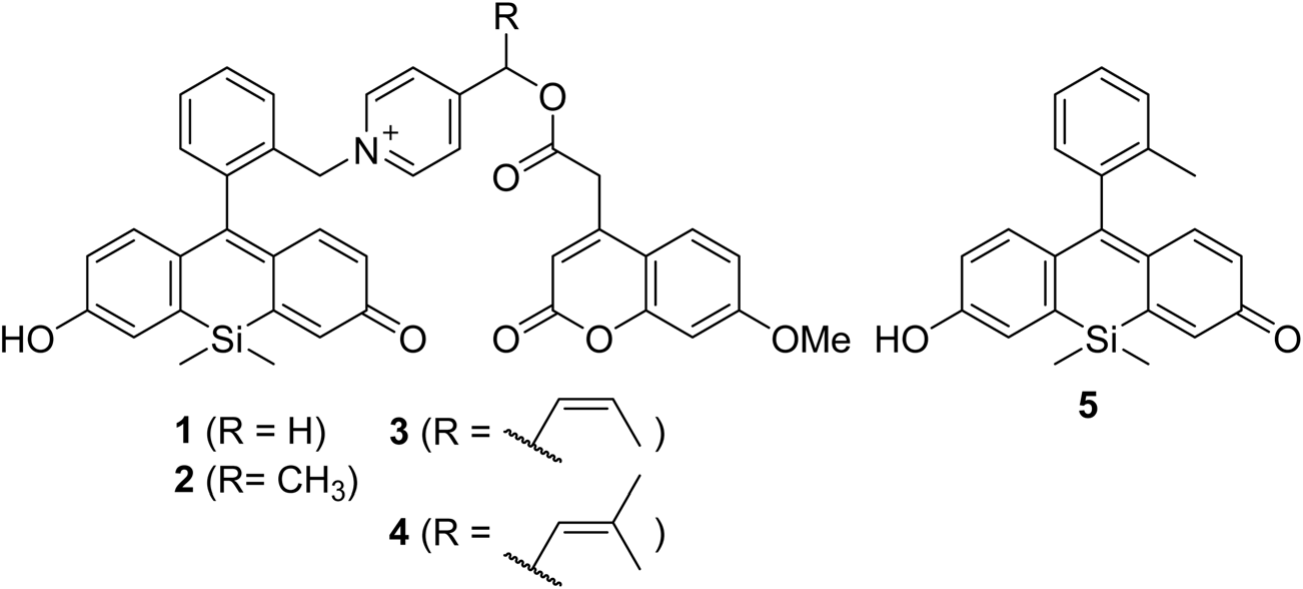
Chemical structures of 7-methoxycoumarin-derived compounds protected with the newly designed orange-light-responsive PPGs (1–4), along with the reference silicon-fluorescein derivative (5).

Based on these predictions, we designed and synthesized compounds 1–4 bearing a protected coumarin derivative, which exhibits strong fluorescence and is readily detectable by HPLC (Fig. 2). Among them, 1 has no TS/IM stabilizing substituent, whereas 2, 3, and 4 carry a methyl, a 2-methylvinyl, and a 2,2-dimethylvinyl group, respectively, introduced at the sp^3^ carbon of the picolinium cation as TS/IM stabilizers. As a reference compound, a simple Si-fluorescein derivative, 5 was also prepared for comparison of the spectroscopic characteristics. Although quantum-chemical calculations suggested that the *trans* isomer corresponding to S3 would be more favorable, with a more negative Δ*G*, we were unable to synthesize the *trans* isomer. Therefore, only the *cis* isomer of the disubstituted alkene was evaluated. Briefly, a protected silicon-fluorescein derivative (S6)^35^ was converted to S7 by consecutive reduction *via* palladium-catalyzed hydrogenation and lithium aluminum hydride treatment (see SI). The hydroxyl group of S7 was substituted with a bromo group by mesylation and subsequent nucleophilic attack using lithium bromide. Removal of two methoxymethyl groups of S7 with trimethylsilyl bromide and oxidation with chloranil resulted in formation of an intermediate S9 which was converted to 1–4 by S_N_2 reaction with S16–19. Compound 5 was prepared based on the previous report.^34^ The structure and purity of these compounds were confirmed by ^1^H NMR, ^13^C NMR, HRMS, and HPLC.

**Fig. 3:**
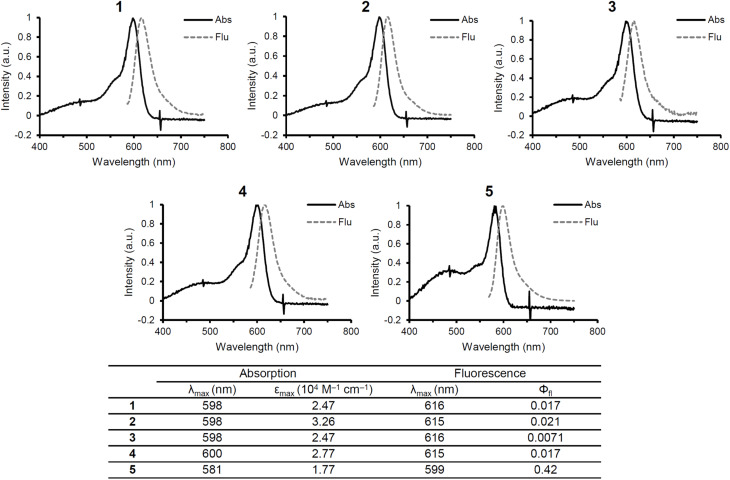
Absorption (solid) and fluorescence (dashed) spectra of compounds 1–5 (10 µM) recorded in 100 mM HEPES buffer (pH 7.3, DMSO 0.1%). The corresponding photophysical properties, including absorption maxima (*λ*_max_), extinction coefficient (*ε*_max_), fluorescence maxima (*λ*_max_), and fluorescence quantum yields (*Φ*_fl_), are summarized in the table. Excitation wavelength: 582 nm. Quantum yields were determined using 5 as a standard.

First, the spectroscopic characteristics of 1–4 were investigated and compared with those of 4. As shown in Fig. 3, all of the compounds exhibited absorption at around 580–600 nm. Si-Fluorescein derivatives are known to absorb near 580 nm in their deprotonated form, suggesting that all of the compounds are deprotonated at pH 7.4. The absorption spectra of 1–4 were red-shifted by 17–19 nm compared to that of 5. As proposed for previous PeT-type caged compounds, this red-shift is likely due to π–π stacking between the antenna moiety and the bond-cleavage site.^19,26^ Compared to the reference compound 5, compounds 1–4 showed decreased fluorescence quantum yields, which is likely attributable to PeT occurring between the antenna moiety and the picolinium cation.

**Fig. 4:**
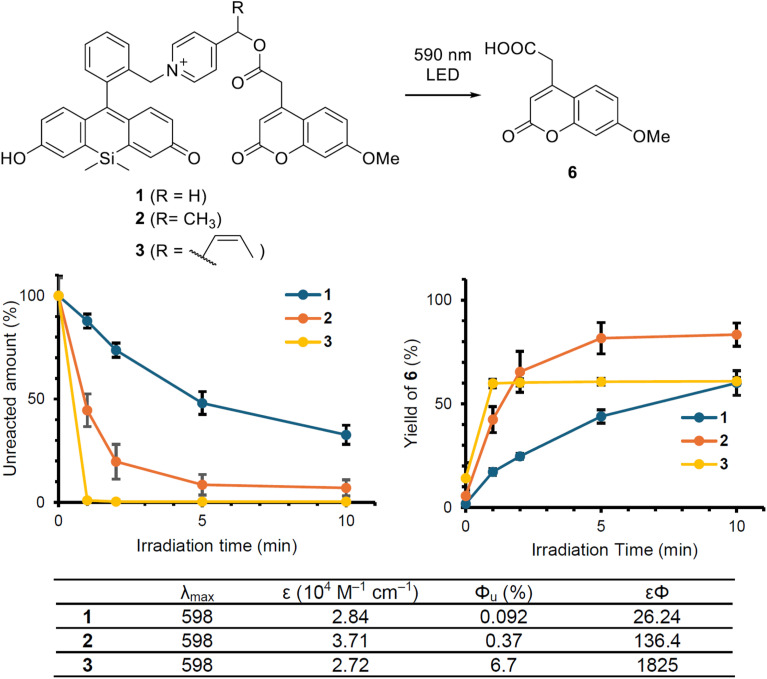
Monitoring the photodecomposition of 1–3 and the photorelease of 6. Time course of the concentrations of compounds 1–3 and the released product 6 during photoirradiation. The table summarizes the photochemical parameters for 1–3, including the extinction coefficient (*ε*), uncaging quantum yield (*Φ*), and the product of *ε* and *Φ* (*εΦ*), representing the overall photorelease efficiency.

To investigate the photodecomposition behavior of the synthesized compounds, we conducted photoirradiation experiments with 1–4. However, compound 4 was found to be the most unstable among the series, undergoing rapid hydrolysis even in the dark, which made accurate measurements difficult (Fig. S3 and S4). This pronounced instability is consistent with the results of quantum-chemical calculations: compound S5, a simplified model corresponding to 4, showed a negative Δ*G* value. Although the computed structure does not fully capture the actual molecular environment of 4, the result suggests that the 2,2-dimethylvinyl group strongly stabilizes the carbocation intermediate that would likely be formed during the undesired hydrolysis. This stabilization may account for the exceptional lability of 4 in the dark. Therefore, we focused on evaluating the photoreactivity of 1–3. To compare the photodecomposition rates of 1–3, a solution of each compound (10 µM) in 100 mM HEPES buffer (pH 7.3, 0.1% DMSO) was photoirradiated with orange light (590 nm LED, 36 mW cm^−2^) and analyzed by HPLC. As shown in Fig. 4, upon investigating the photodegradation of 1–3, we found the reactivity to follow the order 1 < 2 < 3. These results suggest that the substituents on the sp^3^ carbon of the picolinium cation significantly influence the reaction rate. The release rates of 6 also followed the order 1 < 2 < 3, indicating that these compounds undergo the expected bond-cleavage reaction. To quantitatively evaluate these photoreactions, we measured the uncaging quantum yields (*Φ*_u_) of release of 6. A xenon light source (600 nm, bandwidth 5 nm) from a fluorescence spectrometer (RF-5300, Shimadzu) was used for photoexcitation, and the amount of released 6 was quantified by HPLC (Tables S1–3). For the quantum yield measurements, we irradiated 10 µM solutions of each compound with different light doses so that approximately 0.5–1.5 µM of 6 was released. The photouncaging quantum yields were measured using irradiation periods of 30 and 60 seconds, during which spontaneous hydrolysis is negligible. The number of incident photons from the light source was determined using Reinecke's salt (NH_4_[Cr(NCS)_4_(NH_3_)_2_]·1H_2_O) as a chemical actinometer.^40^ The *Φ*_u_ values of 6 release for 1–3 were determined to be 0.092%, 0.37%, and 6.7%, respectively. Furthermore, the reaction efficiencies, considering the extinction coefficients (*εΦ*) for 1–3, were calculated to be 26.2, 136, and 1825, respectively. Among PPGs capable of caging carboxylic acids and being activated by low-toxicity light above 560 nm, the PPG substituted with a 2-methylvinyl group exhibits one of the highest efficiencies reported to date.^15^ These results further support the notion that TS/IM stabilizers—such as the 2-methylvinyl group—can modulate both Δ*E*_a_ and Δ*G* through hyperconjugation and extension of π-conjugation, thereby significantly improving the photoreaction efficiency of PeT-type PPGs.

**Fig. 5:**
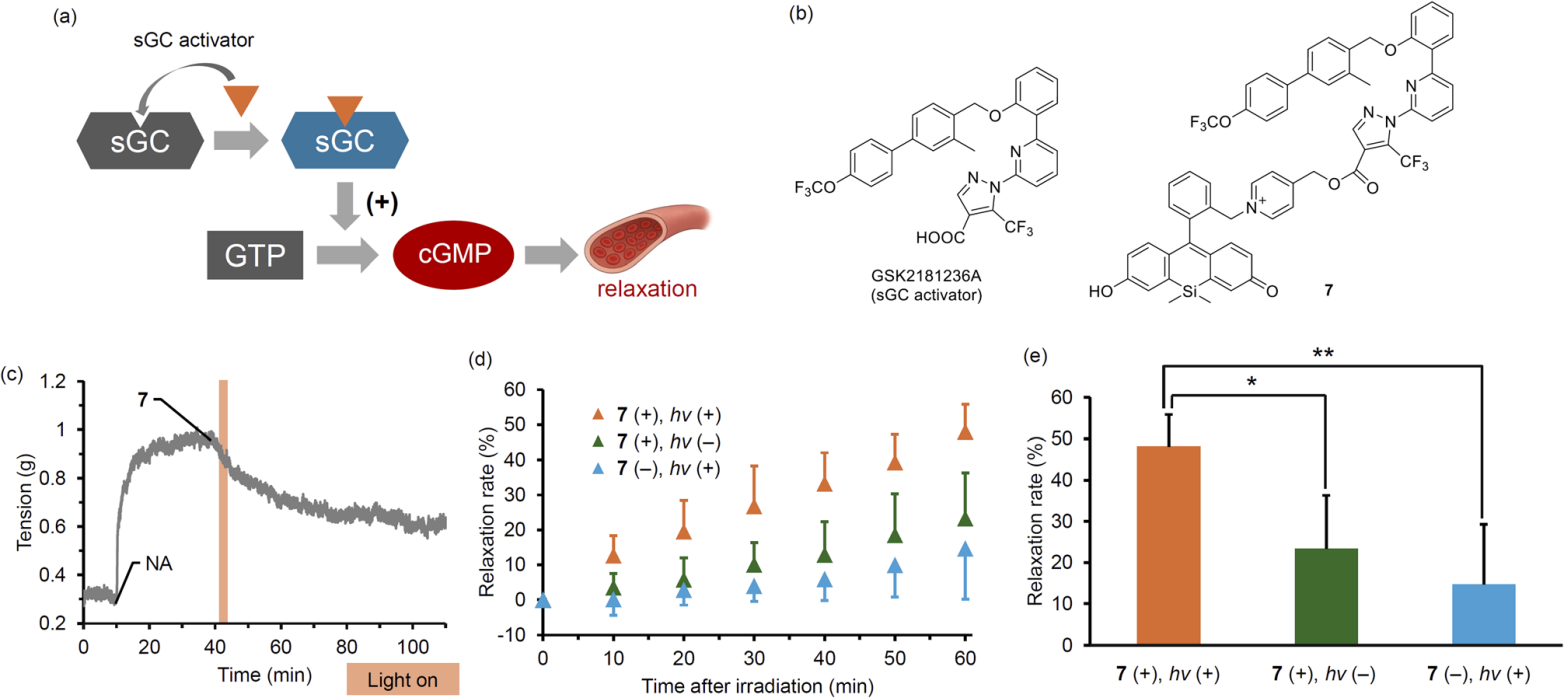
Schematic illustration of the sGC activation pathway (a). Chemical structures of the sGC stimulator GSK2181236A and its protected derivative using the newly developed orange-light-responsive PPG ((b), 7). Changes in the tension of rat aorta *ex vivo* induced by orange-light-mediated drug release from 7 in the presence of a nitric oxide synthase inhibitor, *N*-G-nitro-l-arginine methyl ester (l-NAME, 10 µM). Rat aorta in a glass tube was treated with noradrenaline (NA, 10 µM). The tube was irradiated with a 590 nm LED (c). Time course of relaxation rate after light irradiation under three different conditions. Shown are the vascular relaxation responses following orange-light exposure in the presence of 7 (orange), in the absence of 7 (green), and in the presence of 7 without light irradiation (blue, d). Comparison of relaxation rates at 60 minutes post-irradiation under the same three experimental conditions described above (e). Data are presented as mean ± SE (error bars, *n* = 4). Statistical significance was assessed using Dunnett's test. *p < 0.05, and **p < 0.01.

To demonstrate the applicability of these orange-light-responsive PPGs for the optical control of biological events, we focused on the light-mediated regulation of vasodilation (Fig. 5). Blood flow influences virtually all tissues in the body, and its dysregulation is directly linked to various organ diseases. Thus, the ability to optically induce localized vasodilation may open the door to therapeutic strategies with reduced side effects for a range of diseases.^41,42^ To this end, we focused on GSK2181236A, a known vasodilator possessing a carboxylic acid group and functioning as an activator of soluble guanylate cyclase (sGC).^43^ Although the weak UV absorption of GSK2181236A made it difficult to quantify the photoproducts and determine the quantum yield, we previously demonstrated that this PeT-type PPG acts as an effective cage for carboxylic acids. Given that the carboxyl group of GSK2181236A plays a critical role in binding to sGC, we hypothesized that the biological activity of this compound could be photocontrolled using our PPG strategy. This compound binds to the oxidized or heme-deficient form of sGC and enhances the production of cyclic GMP. We designed and synthesized 7, in which GSK2181236A is protected with one of our newly developed orange-light-responsive PPGs. We evaluated the photovasodilatory effect of 7 on rat aorta under orange-light irradiation. All animal experiments were performed in accordance with the Guiding Principles for the Care and Use of Laboratory Animals of the Science and International Affairs Bureau of the Japanese Ministry of Education, Culture, Sports, Science, and Technology, and were approved by the Animal Experimentation Ethics Committee of Nagoya City University (No. 24-012). We placed a strip of rat aorta in a Magnus tube filled with Krebs buffer, and tensioned it by exposing it to noradrenaline (NA). We also administered a nitric oxide synthase inhibitor, l-NAME, to eliminate the effect of endogenous NO. After the tension had reached a plateau, we conducted orange-light irradiation with a 590 nm LED (30 mW cm^−2^). As shown in Fig. 5c, the smooth muscle tension was decreased after 3 minutes of irradiation, whereas no marked effect was observed without 7 or without irradiation. The slight vasodilation observed in the absence of 7 or light irradiation was probably induced by DMSO, since it has been reported that DMSO induces vasodilation in rat aorta by inhibiting Rho-kinase.^44^ While the vasodilation produced by caged NOs was transient and only occurred during irradiation,^17^7 induced prolonged vasodilation even after the irradiation was stopped. These results demonstrate that 7 can serve as an effective tool for the optical induction of sustained vasodilation using low-toxicity orange light. Given its ability to release a clinically relevant vasodilator with temporal precision and minimal invasiveness, we believe 7 holds great potential for applications in vascular biology research, as well as for the development of novel therapeutic strategies that combine optical control with reduced systemic side effects.

## Conclusion

In this study, we developed a new class of photolabile protecting groups (PPGs) that can be activated by low-toxicity visible light, based on an original molecular design strategy. A key feature of our molecular design is its focus on the post-PeT intermediate. Specifically, we employed quantum-chemical calculations to evaluate Δ*E*_a_ and Δ*G* associated with the bond cleavage step that follows PeT. By optimizing the structure of the PPG moiety to lower the activation barrier of this critical step, we were able to achieve a significant increase in the uncaging quantum yield. The correlation between theoretical predictions and experimental data not only validates our design concept, but also provides a rational framework for future development of more functionalized PPGs. To illustrate the utility of these PPGs, we synthesized a new caged compound in which a vasodilatory drug molecule was protected with our newly developed PPG. Upon visible light irradiation, the compound released the active drug, resulting in sustained vasodilation. This outcome highlights the potential of our system to optically regulate vascular tone using biocompatible wavelengths of light, an approach that minimizes both systemic exposure and off-target effects. The success of this proof-of-concept study not only underscores the advantages of our PPG design but also offers important mechanistic insights into how molecular structure influences PeT-based photochemical reactivity. This knowledge is expected to inform the rational design of next-generation PPGs that are responsive to light of even longer wavelengths, such as near-infrared, which would offer even greater tissue penetration and safety for *in vivo* applications. The development of such light-activated systems could enable localized treatment with high precision, potentially reducing side effects and improving therapeutic outcomes.

In summary, this work presents a rationally designed, visible-light-responsive PPG platform that combines chemical tunability, biological applicability, and theoretical predictability. Our approach lays the groundwork for further development of photoresponsive tools for targeted drug delivery and precise control of physiological processes. We will continue to refine the optical properties and activation efficiencies of these compounds, aiming to extend their applicability in complex biological systems and future clinical settings.

## Author contributions

Conceptualization, N. I. and H. N.; methodology, N. I. and H. N.; investigation, M. T., M. N., Y. H., M. H., K. K., and H. T.; formal analysis, N. I., M. T., M. N., Y. H., and M. H.; data curation, N. I., M. T., M. N., Y. H., M. H., and K. K.; writing – original draft preparation, N. I.; writing – review and editing, N. I., M. N., Y. H., M. H., M. O., and H. N.; visualization, N. I., M. T., M. N., Y. H., and M. H.; supervision, N. I., M. K., and H. N.; project administration, N. I. and H. N.; funding acquisition, N. I. and H. N. All authors have read and agreed to publish this manuscript.

## Conflicts of interest

There are no conflicts to declare.

## Supplementary Material

RA-016-D5RA09470D-s001

## Data Availability

The data supporting this article have been included as part of the supplementary information (SI). Supplementary information: detailed experimental procedures, compound characterization data including NMR spectra, and computational details. See DOI: https://doi.org/10.1039/d5ra09470d.
