# Supplementary material for: Development of silicon-fluorescein-based photolabile protecting groups with enhanced uncaging quantum yield
Source: RSC Adv. 2026 Mar 2;16(13):11964–71. doi: 10.1039/d5ra09470d (PMC12951597; doi:10.1039/d5ra09470d)
Supplement: RA-016-D5RA09470D-s001 [file RA-016-D5RA09470D-s001.pdf]

Supplementary Information (SI) for RSC Advances.
This journal is © The Royal Society of Chemistry 2026

Supporting Information

Development of silicon-fluorescein-based photolabile protecting groups with enhanced uncaging quantum yield

Naoya Ieda,[a,b*] Miyu Tachi,[b] Misuzu Noda,[c] Mei Harada,[a] Yuji Hotta,[c] Kazuki Kondo,[d] Haruka Tsuchiya,[a] Mikako Ogawa,[a,e] Mitsuyasu Kawaguchi,[b] and Hidehiko Nakagawa.[b*]

[a]*Graduate School of Pharmaceutical Sciences, Hokkaido University; N12 W6, Kita-ku Sapporo, Hokkaido, 060-0812, Japan:* [b]*Graduate School of Pharmaceutical Sciences, Nagoya City University; 3-1, Tanabe-dori, Mizuho-ku, Nagoya, Aichi, 467-8603, Japan:* [c]*Graduate School of Medical Sciences, Nagoya City University; 1, Kawasumi, Mizuho-cho, Nagoya, Aichi, 467-8601, Japan:* [d]*WPI-ICReDD, Hokkaido University; N21 W10, Kita-ku, Sapporo, Hokkaido, 001-0021, Japan.*

[*] Correspondence e-mail: ieda@pharm.hokudai.ac.jp, deco@phar.nagoya-cu.ac.jp

General Procedure

Proton nuclear magnetic resonance spectra (^{1}H-NMR) and carbon nuclear magnetic resonance spectra (^{13}C-NMR) were recorded on a JEOL JNM-LA500, JEOL JNM-A500, Varian VNMRS 500 or JEOL JNM-ECZ500 in the indicated solvent. Chemical shifts (δ) were reported in parts per million relative to the internal standard tetramethylsilane. High resolution mass spectra (HRMS) were recorded on a JEOL JMS-SX102A mass spectrometer. Ultraviolet-visible spectra were recorded on an Agilent 8453 spectrophotometer. Fluorescence spectra were recorded on a RF-5300 PC (Shimadzu). Analytical HPLC was performed with a Shimadzu instrument equipped with an Inertsil ODS-3 column (4.6×150 mm, GL Sciences Inc., Japan). Reagents and solvents were purchased from Merck, Tokyo Chemical Industries, FUJIFILM Wako Pure Chemical Corporation, Kanto Chemical, Katayama Chemical, Nacalai Tesque, Junsei Chemical, Kishida Chemical, and Apollo Scientific, and were used without purification. Flash column chromatography was performed using Silica Gel 60 (particle size 0.046–0.063 mm) supplied by Taiko-Shoji. Photoirradiation was performed by using the LED light (CL-1501, Asahi Spectra).

Synthetic scheme for 6–9

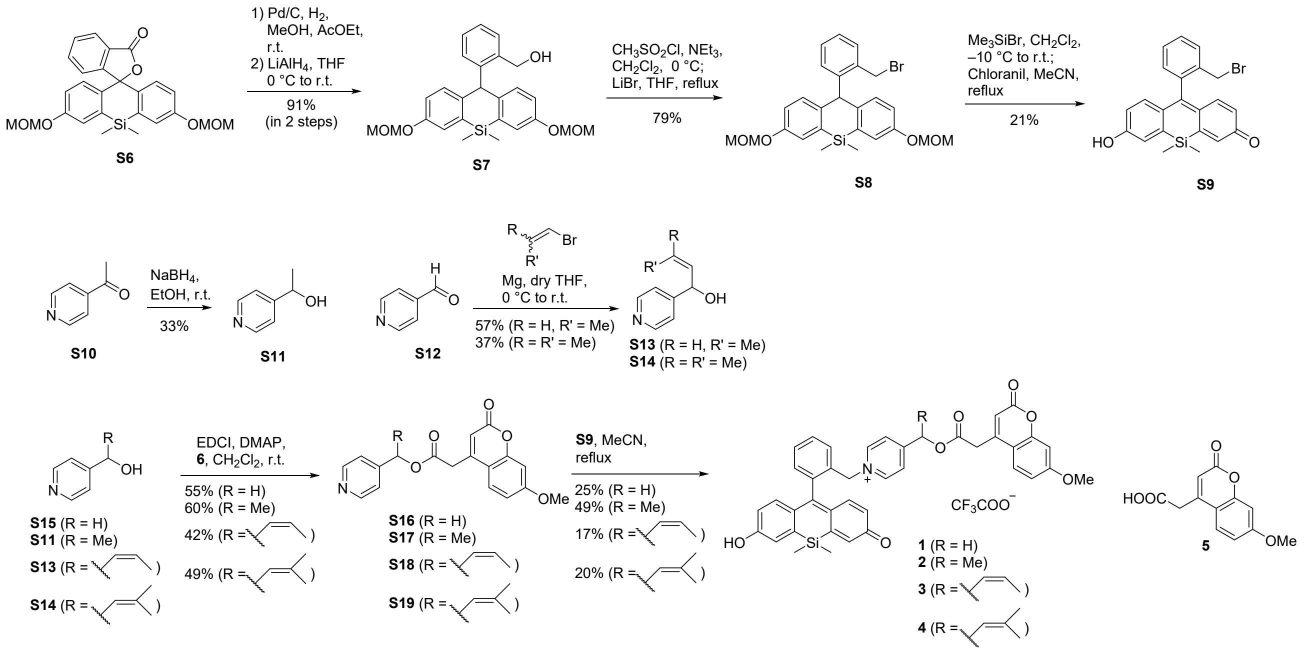

Synthesis of S7

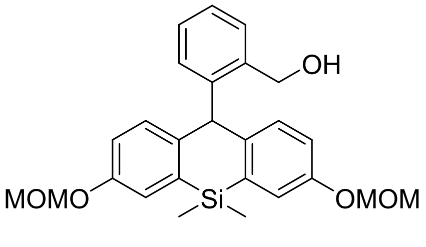

S7

Synthesis of **S7**: **S6** was synthesized based on the reported procedure.[S1] To a solution of **S1** (797 mg, 1.72 mmol, 1.0 equiv.) in a mixture of MeOH and AcOEt (10 mL + 10 mL) was added 10% Pd/C (183 mg, 0.172 mmol, 0.10 equiv.). After stirring at room temperature for 15 h under a hydrogen balloon, the reaction mixture was filtered on Celite to remove Pd/C. The filtrate was concentrated *in vacuo* to obtain a carboxylic acid derivative as a white solid. To a slurry of lithium aluminum hydride (531 mg, 14.0 mmol, 8.0 equiv.) in dry THF (10 mL) was added a solution of the carboxylic acid derivative in dry THF (5+5 mL) on an ice water bath under an argon balloon. After stirring at room temperature for 3.5 h, to the reaction mixture was slowly added water (0.6 mL) followed by 2 N NaOH (1.2 mL), and further water (1.8 mL) on an ice water bath. After stirring on the ice water bath for an hour, the

precipitate was removed by filtration on Celite. The filtrate was concentrated in vacuo and purified by MPLC (*n*-hexane/AcOEt = 73/27 → 52/48) to obtain **S7** (714 mg, 1.58 mmol, 91% in 2 steps) as a clear oil: ^{1}H-NMR ($CDCl_3$, 500 MHz, δ; ppm) 7.41–7.39 (1H, m), 7.25 (2H, d, *J* = 2.7 Hz), 7.21–7.18(2H, m), 7.13–7.12 (1H, m), 7.01 (2H, d, *J* = 8.8 Hz), 6.88 (2H, dd, *J* = 2.8, 8.6 Hz), 5.66 (1H, s), 5.12 (4H, s), 4.56 (2H, s), 3.43 (6H, s), 0.63 (3H, s), 0.43 (3H, s); ^{13}C-NMR($CDCl_3$, 100 MHz, δ; ppm) 154.94, 144.78, 141.75, 137.99, 134.67, 131.53, 130.74, 129.77, 128.20, 126.81, 120.28, 117.41, 94.49, 63.27, 56.03, 50.02, –0.44, –1.26; HRMS (ESI$^+$): cald. 473.17547; found 473.17447 [$(M+Na)^+$] (–1.00 mDa).

Synthesis of S8

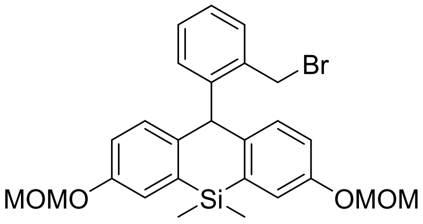

S8

To a solution of **S7** (548 mg, 1.22 mmol, 1.0 equiv.) in CH_2Cl_2 (10 mL) were added triethylamine (340 µL, 2.44 mmol, 2.0 equiv.) and methanesulfonyl chloride (141 µL, 1.82 mmol, 1.5 equiv.) at 0 °C. After stirring at 0 °C for 15 min, the reaction mixture was diluted with CH_2Cl_2 and washed with sat. $NaHCO_3$ aq. twice. The aqueous layer was extracted with CH_2Cl_2 three times. The combined organic layer was washed with brine and dried over Na_2SO_4. Filtration, and evaporation *in vacuo* gave a yellow oil. The residue was dissolved in THF (10 mL) and to the solution was added lithium bromide (314 mg, 3.62 mmol, 3.0 equiv.). After stirring at reflux temperature for an hour, the reaction mixture was quenched with water. After evaporation *in vacuo* to remove THF, the mixture was extracted with CH_2Cl_2 three times. The organic layer was washed with brine and dried over Na_2SO_4. Filtration, and evaporation *in vacuo* gave **S8** (495 mg, 0.964 mmol, 79%) as an orange oil: ^{1}H-NMR ($CDCl_3$, 500 MHz, δ; ppm) 7.46–7.44 (1H, m), 7.25 (2H, d, *J* = 3.1 Hz), 7.20 (2H, dd, *J* = 3.2 Hz, 5.5 Hz), 7.04

(3H, dd, J = 5.7Hz, 9.2 Hz), 6.92 (2H, dd, J = 2.9 Hz, 8.7 Hz), 5.71 (1H, s), 5.16 (4H, s), 4.62 (2H, s), 3.47 (6H, s), 0.63 (3H, s), 0.44 (3H, s); ^{13}C-NMR($CDCl_3$, 100 MHz, δ; ppm) 155.03, 145.38, 141.41, 135.02, 134.91, 131.69, 131.63, 130.86, 129.12, 126.87, 120.35, 117.35, 94.50, 56.03, 49.00, 31.95, –0.59, –1.26; HRMS (ESI$^+$): cald. 535.09107; found 535.09079 [$(M+Na)^+$] (–0.28 mDa).

Synthesis of S9

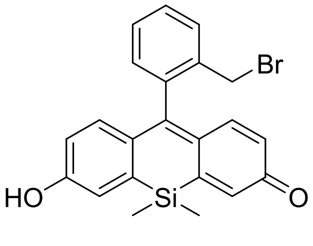

S9

To a solution of **S8** (495 mg, 0.964 mmol, 1.0 equiv.) in dry CH_2Cl_2 (7 mL) was added bromotrimethylsilane (1.25 mL, 9.64 mmol, 10 equiv.) at –10 ºC via syringe pump (0.125 mL/min). After stirring at room temperature for 2 h, the reaction mixture was diluted with CH_2Cl_2 and washed with water three times. The aqueous layer was extracted with CH_2Cl_2. The combined organic layer was washed with brine and dried over Na_2SO_4. After filtration and evaporation *in vacuo*, the residue was dissolved in MeCN (7 mL). To the reaction mixture was added chloranil (711 mg, 2.89 mmol, 3.0 equiv.). After stirring at reflux temperature for 2 h, the reaction mixture was concentrated *in vacuo*. The residue was dissolved in CH_2Cl_2 and the precipitate was removed using a Kiriyama funnel. The filtrate was concentrated *in vacuo* and purified by MPLC (*n*-hexane/AcOEt = 60/40 → 40/60) to obtain **S9** (86 mg, 0.204 mmol, 21%) as a red solid: ^{1}H-NMR ($CDCl_3$, 500 MHz, δ; ppm) 7.59 (1H, d, J = 7.2 Hz), 7.49 (1H, ddd, J = 1.2 Hz, 7.5 Hz, 7.9 Hz), 7.41 (1H, ddd, J = 1.2 Hz, 7.7 Hz, 7.5 Hz), 7.12 (1H, d, J = 6.6 Hz), 7.05 (2H, d, J = 2.3 Hz), 6.88 (2H, d, J = 9.6 Hz), 6.53 (2H, dd, J = 2.4 Hz, 9.3 Hz), 4.21 (2H, s), 0.43 (3H, s), 0.42 (3H, s); HRMS (ESI$^+$): cald. 423.04105; found 423.04070 [$(M+H)^+$] (–0.35 mDa).

Synthesis of **S11**

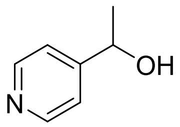

S11

To a solution of **S10** (551 µL, 5.00 mmol, 1.0 eq.) in EtOH (5 mL) was added $NaBH_4$ (232 mg, 6.13 mmol, 1.2 eq.). After stirring at room temperature for 2.5 h, the reaction was quenched with water and the mixture was extracted with DCM three times. The combined organic layer was washed with brine, and dried over Na_2SO_4. Filtration, evaporation *in vacuo* and purification by MPLC (DCM/MeOH = 97/3→90/10→85/15) and gave **S11** (202 mg, 1.64 mmol, 33%) as a clear oil: ^{1}H-NMR ($CDCl_3$, 500 MHz, δ; ppm) 8.49 (2H, dd, J = 1.4 Hz, 3.0 Hz), 7.31–7.30 (2H, m), 4.90 (1H, q, J = 6.5 Hz), 3.47 (1H, s), 1.49 (1H, d, J = 6.8 Hz); ^{13}C-NMR($CDCl_3$, 100 MHz, δ; ppm) 155.22, 149.59, 120.49, 68.67, 25.10; HRMS (ESI$^+$): cald. 124.07569; found 124.07563 [M$^+$] (–0.06 mDa).

Synthesis of **S13**

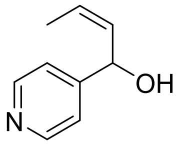

S13

A mixture of magnesium turnings (202 mg, 8.31 mmol, 5.0 eq.) and iodine crystals was heated under an argon balloon until a purple vapor was observed. The reaction mixture was allowed to warm to room temperature, then THF (10 mL) was added followed by 1-bromo-1-propene (558 µL, 6.60 mmol, 4.0 eq.). The mixture was stirred at 40 °C for 15 min, and at room temperature for 2 h. In another two-necked flask, **S12** (354 µL, 3.75 mmol, 1.0 eq.) was dissolved in dry THF (20 mL) under an argon balloon. The mixture was cooled on an ice water bath, and the prepared Grignard reagent solution (0.65 mol/L, 7.00 mL, 4.55 mmol, 1.2 eq.) was slowly added. After stirring on ice water bath for 10 min, the mixture was allowed to warm to room temperature and stirred for 19 h. Then, sat. NH_4Cl aq.

was added, and the aqueous layer was extracted with AcOEt three times. The combined organic layer was washed with brine and dried over Na_2SO_4. Filtration, evaporation *in vacuo*, and purification by MPLC (CH_2Cl_2/CH_3OH = 96/4) gave **S13** (319 mg, 2.14 mmol, 57%) as a purple solid: ^{1}H-NMR ($CDCl_3$, 500 MHz, δ; ppm) 8.57 (2H, dd, *J* = 1.6 Hz, 4.5 Hz), 7.31 (2H, dd, *J* = 1.6 Hz, 4.7 Hz), 5.78–5.74 (1H, m), 5.60–5.54 (2H, m), 1.84 (3H, dd, *J* = 1.6 Hz, 7.3 Hz); ^{13}C-NMR($CDCl_3$, 100 MHz, δ; ppm)152.97, 149.64, 131.93, 127.81, 120.97, 67.89, 13.58; HRMS (ESI$^+$): cald. 150.09134; found 150.09124 [$(M+H)^+$] (–1.66 mDa).

Synthesis of **S14**

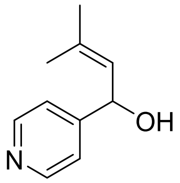

S14

A mixture of magnesium turnings (141 mg, 5.80 mmol, 4.0 eq.) and iodine crystals was heated under an argon balloon until a purple vapor was observed. The mixture was allowed to warm to room temperature, then dry THF (5.2 mL) was added, followed by the addition of 1-bromo-2-methyl-1-propene (451 µL, 4.40 mmol, 3.0 eq.). The mixture was stirred at reflux temperature for 4 h. In another two-necked flask, **S12** (139 µL, 1.47 mmol, 1.0 eq.) was dissolved in dry THF (11 mL) under an argon balloon. The mixture was cooled on an ice water bath, followed by slowly adding the prepared Grignard reagent (0.85 mol/L, 1.90 mL, 1.1 eq.). After stirring on ice water bath for 10 min, the mixture was allowed to warm to room temperature and stirred for 18 h. Then, sat. NH_4Cl aq. was added, and the aqueous layer was extracted with AcOEt three times. The organic layer was washed with brine and dried over Na_2SO_4. Filtration, evaporation *in vacuo*, and purification by MPLC (CH_2Cl_2/MeOH = 96/4) gave **S14** (89.2 mg, 0.547 mmol, 37%) as a white solid: ^{1}H-NMR ($CDCl_3$, 500 MHz, δ; ppm) 8.41 (2H, d, *J* = 4.8 Hz), 7.31 (2H, d, *J* = 6.2 Hz), 5.44 (1H, d, *J* = 9.0 Hz), 5.30–5.29 (1H, m), 1.79 (3H, s), 1.75 (3H, s); ^{13}C-NMR($CDCl_3$, 100 MHz, δ; ppm)153.26, 149.73, 137.00, 126.68, 120.89,

69.35, 25.91, 18.52; HRMS (ESI$^+$): cald. 164.10699; found 164.10684 [$(M+H)^+$] (–0.15 mDa).

Synthesis of **S16–19**

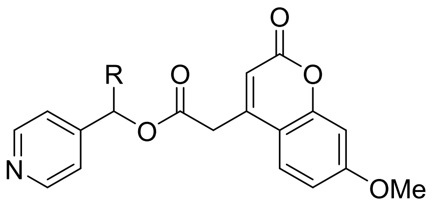

S16 (R = H)
S17 (R = Me)
S18 (R = 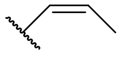)
S19 (R = 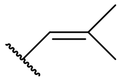)

To a mixture of an alcohol derivative (**S11**, **S13–15**, 1.0 equiv.), **6** (1.2 eq.) and *N*,*N*-dimethyl-4-aminopyridine (0.85 eq.) in CH_2Cl_2 (0.2 mol/L) was added EDCI (1.2 eq.). The reaction was monitored by TLC to confirm completion. The reaction was quenched with water and the mixture was extracted with CH_2Cl_2 three times. The combined organic layer was washed with brine and dried over Na_2SO_4. Filtration, evaporation *in vacuo* and purification by MPLC (CH_2Cl_2/MeOH = 90/10) gave the ester derivative (42–60%).

S16: ^{1}H-NMR ($CDCl_3$, 500 MHz, δ; ppm) 8.58 (2H, d, J = 6.0 Hz), 7.44 (1H, d, J = 8.7 Hz), 7.16 (2H, d, J = 6.3 Hz), 6.86–6.82 (2H, m), 6.26 (1H, s), 5.17 (2H, s), 3.88 (3H, s), 3.82 (2H, s); ^{13}C-NMR($CDCl_3$, 100 MHz, δ; ppm)168.39, 163.10, 160.75, 155.71, 150.27, 147.58, 143.90, 125.47, 122.05, 114.09, 112.72, 112.32, 101.30, 65.45, 55.93, 38.16; HRMS (ESI$^+$): cald. 326.10230; found 326.10178 [$(M+H)^+$] (–0.52 mDa).

S17: ^{1}H-NMR ($CDCl_3$, 500 MHz, δ; ppm) 8.54 (2H, dd, J = 1.7 Hz, 4.6 Hz), 7.43–7.41 (1H, m), 7.15 (2H, dd, J = 1.7 Hz, 4.5 Hz), 6.87–6.78 (2H, m), 6.25 (1H, s), 5.86 (1H, q, J = 6.7 Hz), 3.86 (3H, s), 3.82 (2H, s), 1.52 (3H, d, J = 6.5 Hz); ^{13}C-NMR($CDCl_3$, 100 MHz, δ; ppm) 167.84, 163.00, 160.70, 155.61, 150.21, 149.40, 147.74, 125.46, 120.56, 113.91, 112.53, 112.28, 101.23, 72.28, 55.84, 38.41, 21.70; HRMS (ESI$^+$): cald. 340.11795; found 340.11736 [$(M+H)^+$] (–0.59 mDa).

S18: ^{1}H-NMR ($CDCl_3$, 500 MHz, δ; ppm) 8.54 (2H, d, J = 5.8 Hz), 7.43 (1H, d, J =8.7 Hz), 7.14 (2H,

d, *J* = 6.1 Hz), 6.88–6.79 (2H, m), 6.58 (1H, d, *J* = 9.3 Hz), 6.26 (1H, s), 5.85–5.79 (1H, m), 5.51–5.47 (1H, m), 3.87 (3H, s), 3.82 (2H, s), 1.82 (3H, dd, *J* = 1.8 Hz, 6.9 Hz); ^{13}C-NMR ($CDCl_3$, 100 MHz, δ; ppm)167.79, 163.01, 160.76, 155.61, 150.16, 147.86, 132.58, 130.87, 127.46, 126.62, 125.55, 120.98, 113.90, 112.58, 101.22, 71.25, 56.89, 38.34, 13.69; HRMS (ESI$^+$): cald. 366.13360; found 366.13322 [$(M+H)^+$] (–0.38 mDa).

S19: ^{1}H-NMR ($CDCl_3$, 500 MHz, δ; ppm) 8.52 (2H, s), 7.41 (1H, d, *J* = 8.7 Hz), 7.12 (2H, d, *J* = 4.1 Hz), 6.82–6.79 (2H, m), 6.49 (1H, d, *J* = 9.3 Hz), 6.25 (1H, s), 5.26 (1H, d, *J* = 8.2 Hz), 3.87–3.86 (3H, m), 3.81 (2H, s), 1.82 (3H, s), 1.76 (3H, s); ^{13}C-NMR($CDCl_3$, 100 MHz, δ; ppm) 167.79, 162.93, 160.75, 155.56, 149.96, 148.44, 147.85, 140.16, 126.78, 126.43, 125.49, 121.33, 120.96, 113.87, 112.50, 112.28, 101.12, 72.73, 55.82, 38.40, 25.84, 18.65; HRMS (ESI$^+$): cald. 380.14925; found 380.14859 [$(M+H)^+$] (–0.66 mDa).

Synthesis of **1**–**4**

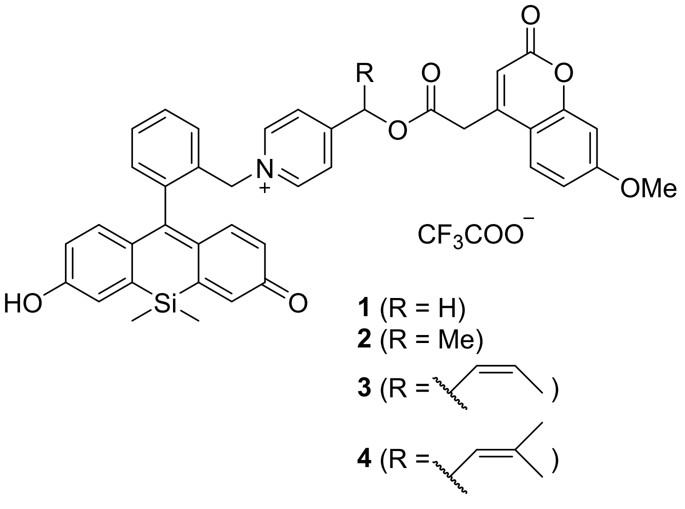

To a solution of **S9** in MeCN (0.01–0.02 mol/L) was added **S16**–**19** (1.9–2.5 equiv.). After stirring at reflux for 1–3 days, the mixture was concentrated *in vacuo*. The residue was purified by preparative HPLC to obtain the picolinium cation derivative (17–49%) as a red solid.

1: ^{1}H-NMR (CD_3CN, 500 MHz, δ; ppm) 8.28 (2H, d, *J* = 6.6 Hz), 7.80–7.78 (1H, m), 7.72–7.67 (2H, m), 7.65–7.63 (1H, m), 7.53 (2H, d, *J* = 6.4 Hz), 7.30–7.28 (1H, m), 7.02 (2H, d, *J* = 2.3 Hz), 6.94–

6.92 (2H, m), 6.46 (2H, d, J = 9.5 Hz), 6.29 (1H, s), 6.22 (2H, dd, J = 2.2 Hz, 9.5 Hz), 5.44 (2H, s), 5.19 (2H, s), 4.01 (2H, s), 3.88 (3H, s), 0.60 (3H, s), 0.42 (3H, s); ^{13}C-NMR (CD_3CN, 125 MHz, δ; ppm) 169.36, 163.91, 161.39, 157.27, 156.47, 152.83, 149.35, 145.16, 144.76, 141.79, 138.64, 133.18, 131.69, 131.53, 131.31, 130.59, 130.34, 127.29, 125.94, 123.31, 114.57, 114.56, 113.35, 113.05, 101.98, 64.46, 63.68, 56.67, 56.66, 37.74, –0.77, –1.68; HRMS (ESI$^+$): calcd: 668.21045; found: 668.21158 [M$^+$] (+1.13 mDa). Purity by reverse-phase HPLC was 97.4 % (254 nm).

2: ^{1}H-NMR (CD_3CN, 500 MHz, δ; ppm) 8.28 (2H, d, J = 6.7 Hz), 7.80 (1H, d, J = 8.0 Hz), 7.73–7.67 (2H, m), 7.55–7.54 (3H, m), 7.28 (1H, dd, J = 1.8 Hz, 6.9 Hz), 7.01 (2H, dd, J = 2.4 Hz, 6.0 Hz), 6.91–6.88 (2H,m), 6.52–6.46 (2H, m), 6.24–6.19(3H, m), 5.82 (1H, q, J = 6.7 Hz), 5.42 (2H, s), 3.92 (2H, d, J = 2.3 Hz), 3.86 (3H, s), 1.36 (1H, d, J = 6.6 Hz), 0.58 (3H, s), 0.43 (3H, s); ^{13}C-NMR (CD_3CN, 125 MHz, δ; ppm) 169.08, 163.88, 161.96, 161.36, 156.45, 149.41, 145.41, 141.88, 138.80, 138.66, 133.37, 131.75, 131.57, 131.12, 130.49, 130.42, 130.36, 127.26, 125.35, 123.39, 123.14, 114.57, 114.56, 113.32, 113.02, 101.96, 71.70, 63.67, 56.67, 38.05, 21.64, –0.82, –1.45;HRMS (ESI+): calcd: 682.22610; found: 682.22828 [M$^+$] (+2.17 mDa). Purity by reverse-phase HPLC was 99.0 % (254 nm).

3: ^{1}H-NMR (CD_3CN, 500 MHz, δ; ppm) 8.26 (2H, d, J = 6.8 Hz), 7.80 (1H, dd, J = 2.0 Hz, 9.0 Hz), 7.67–7.73 (2H, m), 7.55 (1H, d, J = 8.8 Hz), 7.50 (2H, d, J = 6.7 Hz), 7.27 (1H, dd, J = 1.8 Hz, 8.7 Hz), 7.00 (2H, dd, J = 2.0 Hz), 6.93–6.89 (2H, m), 6.51 (2H, dd, J = 2.0 Hz, 7.5 Hz), 6.47 (1H, d, J = 9.4 Hz), 6.25–6.18 (3H, m), 5.92–5.89 (1H, m), 5.42 (2H, s), 5.20–5.15 (1H, s), 3.95 (2H, s), 3.87 (3H, s), 1.79 (3H, dd, J = 1.7 Hz, 5.2 Hz), 0.57 (3H, s), 0.43 (3H, s); ^{13}C-NMR (CD_3CN, 125 MHz, δ; ppm) 168.70, 163.94, 161.39, 159.98, 156.50, 153.36, 149.38, 145.43, 145.05, 141.71, 138.90, 138.82, 133.95, 133.40, 131.84, 131.63, 131.12, 130.84, 130.44, 127.17, 125.63, 125.57, 123.24, 123.17, 114.53, 113.29, 113.06, 102.01, 70.85, 63.75, 56.69, 38.10, 13..96, –0.86, –1.55; HRMS (ESI$^+$): calcd: 708.24175; found: 708.24582 [M$^+$] (+4.06 mDa).; Purity by reverse-phase HPLC was 98.1% (254 nm).

4: ^{1}H-NMR (CD_3CN, 500 MHz, δ; ppm) 8.25 (2H, d, J = 6.6 Hz), 7.79 (1H, dd, J = 1.6Hz, 6.9 Hz), 7.73–7.68 (2H, m), 7.54 (1H, d, J = 8.8 Hz), 7.49 (2H, d, J = 6.4 Hz), 7.28 (1H, dd, J = 1.5 Hz, 6.8

Hz), 7.00 (2H, dd, J = 2.7 Hz), 6.92–6.89 (2H, m), 6.50 (2H, dd, J = 9.6 Hz), 6.43 (1H, d, J = 9.4 Hz), 6.26–6.19 (3H, m), 5.42 (2H, s), 4.95 (1H, d, J = 9.5 Hz), 3.93 (2H, s), 3.87 (3H, s), .1.79 (6H, dd, J = 1.1 Hz, 16.8 Hz), 0.57 (3H, s), 0.43 (3H, s); ^{13}C-NMR (CD_3CN, 125 MHz, δ; ppm) 168.79, 163.95, 161.40, 160.50, 156.50, 153.37, 149.47, 145.32, 145.11, 145.02, 143.90, 141.71, 138.87, 133.38, 131.84, 131.63, 131.16, 130.90, 130.81, 130.45, 127.18, 125.66, 120.15, 114.50, 113.31, 113.01, 102.02, 72.27, 63.73, 56.70, 38.20, 25.82, 18.95, –0.83, –1.56; HRMS (ESI$^+$): calcd: 722.25740; found: 722.26123 [M^+] (+3.83 mDa).; Purity by reverse-phase HPLC was 97.2 % (254 nm).

Synthetic scheme for 7

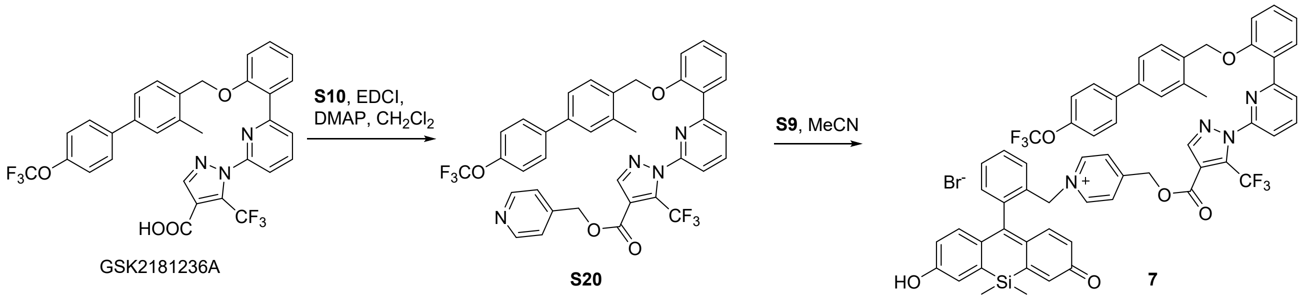

Synthesis of **S20**

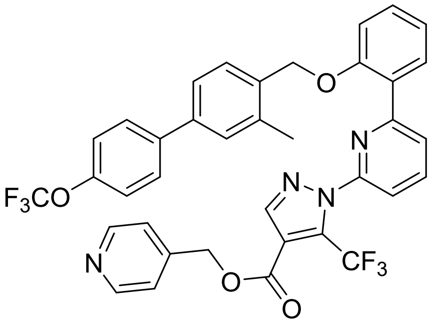

S20

To a solution of GSK2181236A (307 mg, 0.500 mmol, 1.0 equiv.), **S15** (65 mg, 0.596 mmol, 1.2 equiv.), and 4-*N,N*-dimethyaminopyridine (61 mg, 0.499 mmol, 1.0 equiv.) in CH_2Cl_2 (10 mL) was added EDCI (105 mg, 0.548 mmol, 1.1 equiv.). After stirring at room temperature for an hour, the reaction mixture was poured into sat. NH_4Cl (50 mL) and water (50 mL) and extracted with CH_2Cl_2 three times. The organic layer was washed with brine and dried over Na_2SO_4. Filtration, evaporation *in vacuo*, and purification by MPLC (CH_2Cl_2/MeOH = 95/5 → 90/10 → 85/15) gave **S20** (68%) as a clear oil: ^{1}H-NMR (CD_3OD, 500 MHz, δ; ppm) 8.56 (2H, d, *J* = 6.3 Hz), 8.28 (1H, s), 8.10 (1H, d, *J* = 8.1 Hz), 7.97 (1H, dd, *J* = 8.0 Hz, 8.0 Hz), 7.83 (1H, dd, *J* = 1.7 Hz, 7.7 Hz), 7.68 (2H, d, *J* = 8.6 Hz), 7.61 (1H, d, *J* = 8.0 Hz), 7.51 (2H, d, *J* = 6.3 Hz), 7.46–7.41 (4H, m), 7.32 (2H, d, *J* = 8.0 Hz), 7.28 (1H, d, *J* = 8.1 Hz), 7.11 (1H, dd, *J* = 7.4 Hz, 7.4 Hz), 5.44 (2H, s), 5.23 (2H, s), 2.34 (3H, s); ^{13}C-NMR($CDCl_3$, 100 MHz, δ; ppm) 160.71, 156.59, 156.55, 155.20, 151.01, 150.26, 148.76, 144.36, 142.68, 139.72, 139.55, 138.63, 136.89, 134.16, 130.95, 129.20, 128.84, 128.47, 127.63, 127.58, 126.15, 124.75, 122.03, 121.88, 121.34, 116.84, 116.65, 113.32, 69.13, 65.18, 19.12; HRMS (ESI$^+$): cald. 705.19310; found 705.19213 [$(M+H)^+$] (–0.97 mDa).

Synthesis of **7**

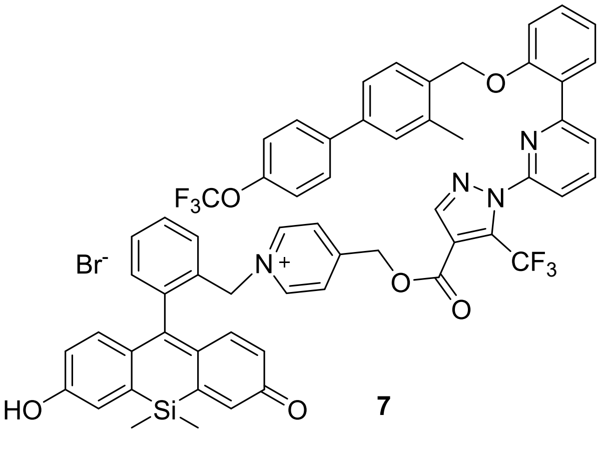

7

A solution of **S9** (114 mg, 0.340 mmol, 1.0 equiv.), and **S20** (239 mg, 0.339 mmol, 1.0 equiv.) in MeCN (10 mL) was stirred at reflux temperature for 14 h. After evaporation, the residue was recrystallized with MeCN/Et_2O to obtain **7** (16%) as a red solid: ^{1}H NMR (CD_3OD, 500 MHz, δ; ppm) 8.62 (2H, d, *J* = 6.6 Hz), 8.37 (1H, s), 8.13 (1H, dd, *J* = 1.0 Hz, 8.0 Hz), 8.01 (1H, dd, *J* = 8.0 Hz, 8.0 Hz), 7.86–7.81 (4H, m), 7.77–7.69 (4H, m), 7.64 (1H, d, *J* = 8.1 Hz), 7.48–7.41 (4H, m), 7.34–7.30 (4H, m), 7.11 (1H, ddd, *J* = 0.9 Hz, 7.5 Hz, 7.5 Hz), 7.03 (2H, s), 6.61 (2H, d, *J* = 9.4 Hz), 6.28 (2H, d, *J* = 7.4 Hz), 5.61 (2H, s), 5.51 (2H, s), 5.25 (2H, s), 2.35 (3H, s), 0.63 (3H, s), 0.45 (3H, s); ^{13}C NMR (CD_3OD, 125 MHz, δ; ppm) 161.03, 158.11, 157.94, 156.67, 155.71, 152.22, 149.91, 149.90, 145.92, 143.99, 141.98, 141.14, 140.78, 140.24, 138.59, 135.70, 134.13, 133.80, 133.42, 132.10, 131.89, 131.84, 131.00, 130.31, 129.93, 129.56, 128.77, 127.53, 126.23, 125.54, 122.98, 122.41, 122.36, 121.74, 120.94, 119.59, 118.45, 117.16, 114.73, 70.20, 64.83, 64.06, 19.07, –0.82, –1.66; HRMS (ESI$^+$): cald. 1047.30125; found 1047.29959 [M$^+$] (–1.66 mDa); Anal. Calcd. for $C_{59}H_{45}BrF_6N_4O_6Si$•3/2$H_2O$: C, 61.35; H, 4.19; N, 4.85. Found: C, 61.14; H, 4.22; N, 4.96.

Quantum chemical calculation

All calculations were carried with the Gaussian 16 program.[S2] The molecular structure optimizations were conducted at the UωB97X-D level using the cc-pVDZ basis set. Solvation was evaluated by the

self-consistent reaction field (SCRF) method using the polarizable continuum model (PCM, SOLVENT = Water). The intrinsic reaction coordinate (IRC) method was used to track minimum energy paths from transition structures to the corresponding local minima.[S3] In this study, the Gibbs free energy was adopted as the basis for discussion. All the stationary structures have no imaginary frequencies and the TS structures have one imaginary frequency.

Measurement of absorption and fluorescence spectra

Absorption and fluorescence spectra were measured using a UV-Vis spectrophotometer (Agilent 8453) and a fluorescence spectrophotometer (RF5300-PC), respectively. Fluorescence spectra were recorded with an excitation wavelength of 582 nm. The fluorescence quantum yield was calculated relative to compound **10** (1 μM in 100 mM sodium phosphate buffer, pH 9.0, DMSO 1%) as a standard (Φ_{fl} = 0.42)

HPLC analysis of photolysis

A solution of each compound (10 μM) in 100 mM HEPES buffer (pH 7.3, DMSO 0.1%, 10 mL) was irradiated by a 590 nm LED (41 mW/cm^2) at 37 ºC. After irradiation, an aliquot was subject to HPLC performed with a Shimadzu instrument equipped with an Inertsil ODS-3 column (4.6 × 150 mm, GL Science Inc.). HPLC conditions were as follows: solvent A, MilliQ (0.1% TFA); solvent B, MeCN (0.1% TFA); B conc., 5 to 100% (20 min) → 100 to 100% (25 min) → 100 to 5% (26 min) → 5 to 5% (30 min).

Calculation of uncaging quantum yields

Measurement of the amount of **6** released during irradiation: A solution of each compound (10 μM) in 100 mM HEPES buffer (pH 7.3, 0.1% DMSO, total volume: 3 mL) was irradiated at 600 nm (**1**: bandwidth = 20 nm, for 90 s; **2**: bandwidth = 20 nm, for 30 s; **3**: bandwidth = 5 nm, for 60 s) with the Xe lamp of a fluorescence spectrometer, RF5300 (Shimadzu) at 37 °C. After irradiation, an aliquot

was subjected to HPLC on a Shimadzu instrument equipped with an Inertsil ODS-3 column (4.6 × 150 mm, GL Science Inc., Japan). HPLC conditions were as follows: solvent A, MilliQ (0.1% TFA); solvent B, MeCN (0.1% TFA); B conc., 5 to 100% (20 min) → 100 to 100% (25 min) → 100 to 5% (26 min) → 5 to 5% (30 min). The excitation wavelength for **6** was 325 nm.

Measurement of the number of photons: The number of photon was determined using $NH_4[Cr(NH_3)_2(SCN)_4]$ (Reinecke's salt) as a chemical actinometer. Reinecke's salt absorbs visible light and undergoes a photochemical ligand-exchange reaction with water, releasing thiocyanate ions (eq. S1). The liberated thiocyanate was trapped with ferric ions to form the iron(III) thiocyanate complex, and its concentration was quantified by measuring the absorbance (eq. S2). The quantum yield of this ligand-exchange reaction has been reported to be 0.28, and this value was used to calculate the incident photon flux (irradiated light dose).

$$[Cr(NH_3)_2(SCN)_4]^- + H_2O \rightarrow [Cr(NH_3)_2(SCN)_3(H_2O)]^- + SCN^- \text{ (eq. S1)}$$

$$Fe^{3+} + SCN^- \rightarrow Fe(SCN)^{2+} \text{ (eq. S2)}$$

An aqueous solution (3 mL) of Reinecke's salt (10 mM) was irradiated under the same conditions as described above. After irradiation, an aliquot (50 µL) was mixed with 400 µL of an aqueous solution containing 0.5 M $HClO_4$ and 0.1 M $Fe(NO_3)_3$ and 400 µL of MilliQ water. The absorption spectrum of the mixture was recorded on an Agilent 8453 spectrometer. The absorption spectrum of the non-irradiated mixture was also recorded. The photon number was calculated from the absorption difference (λ_{max} = 450 nm, ε = 4300 L mol^{-1} cm^{-1}). The calculations were performed with reference to the quantum yield of Reinecke's salt for thiocyanate anion release (Φ = 0.28).

Photomanipulation of rat aorta strip

An aortic strip from an 11-week-old male SD rat was placed in a Magnus tube filled with Krebs buffer at 37 °C. The tension was recorded on a LabChart7 (ADInstruments). The strip was pre-treated with L-NAME (10 µM). Pre-contraction was induced by noradrenaline (10 µM). After equilibration, **7** (10

μM), or DMSO (100 μL) was added, and the tube was irradiated with a CL-1501 lamp (Asahi Spectra) with a 590 nm LED head (30 mW cm^{-2}) for 3 min.

NMR Charts

Compound 1

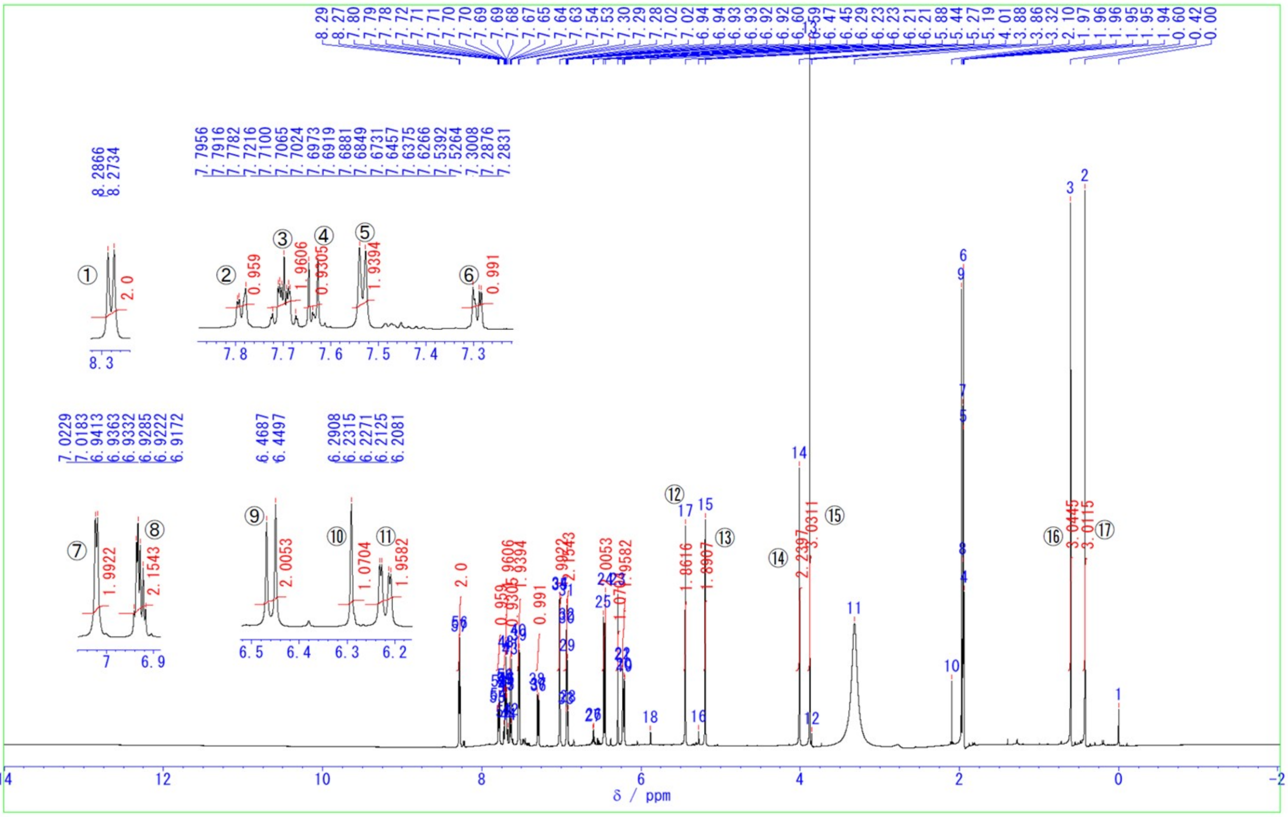

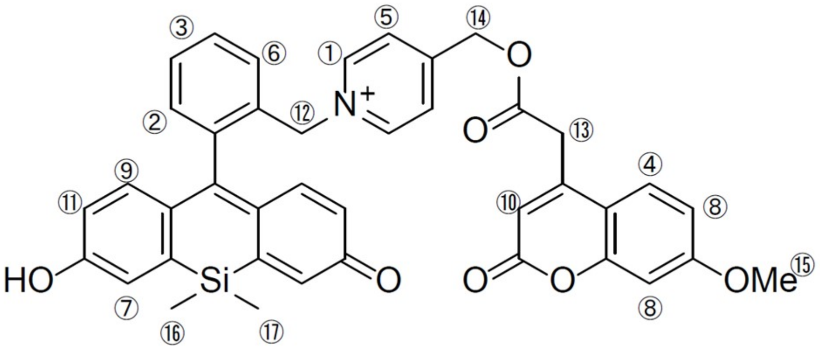

Compound 2

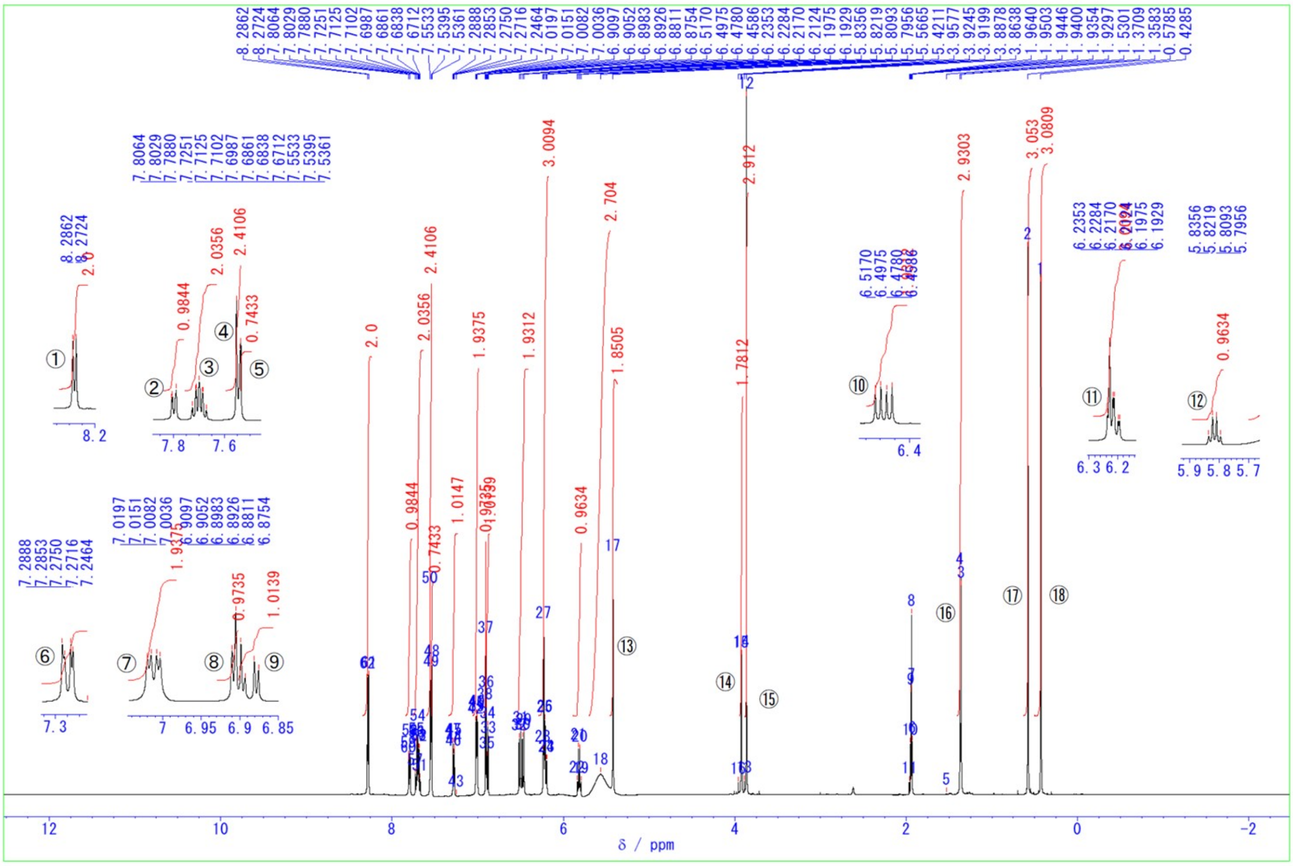

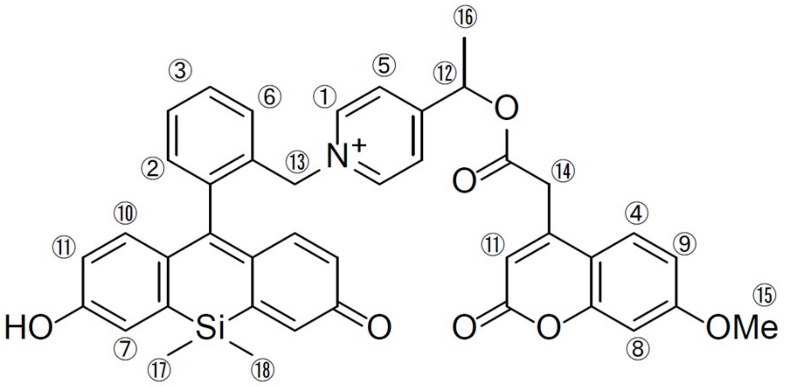

Compound 3

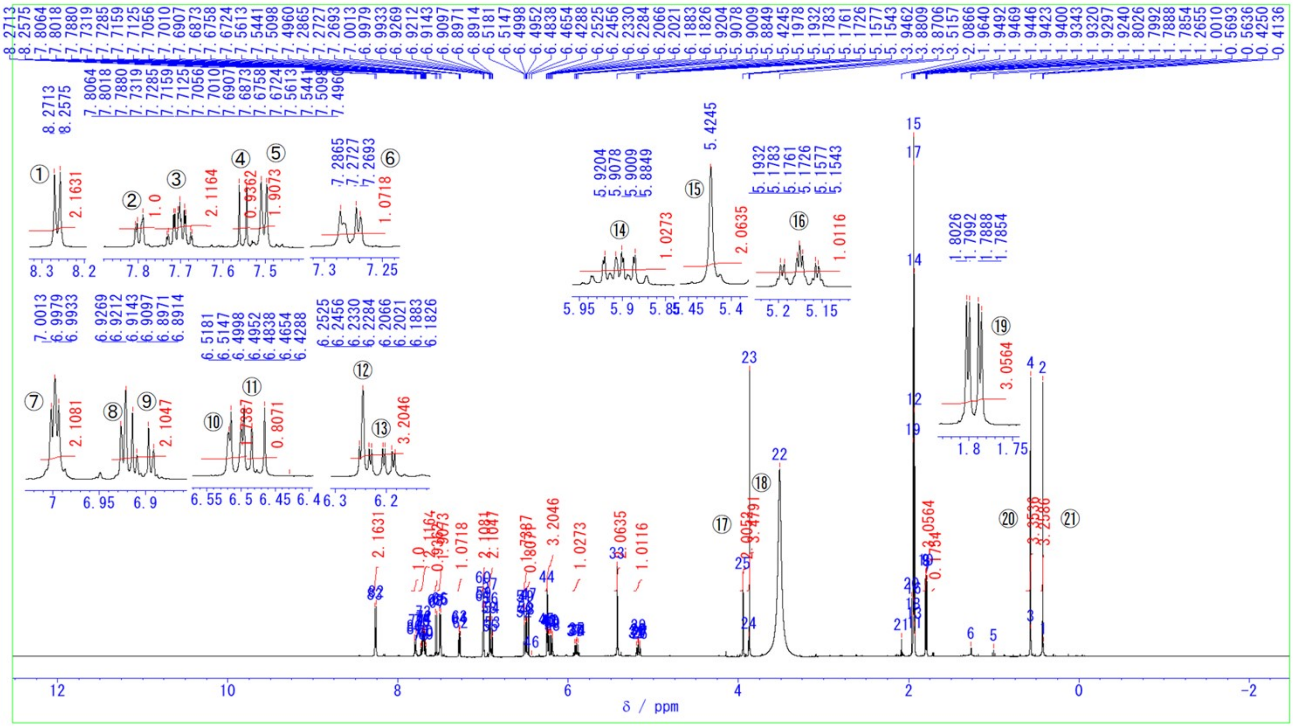

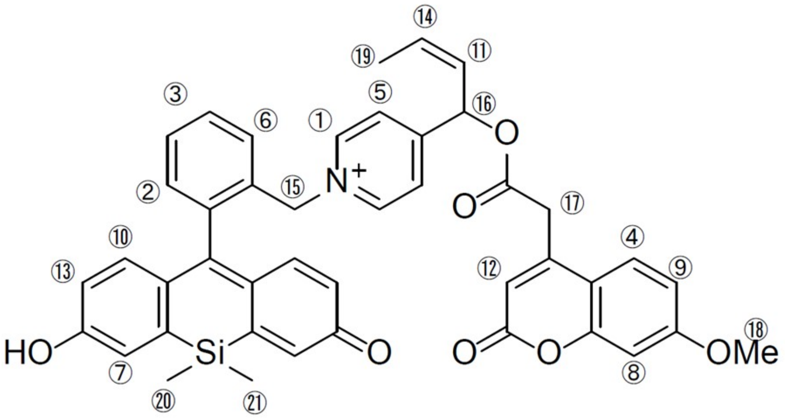

Compound 4

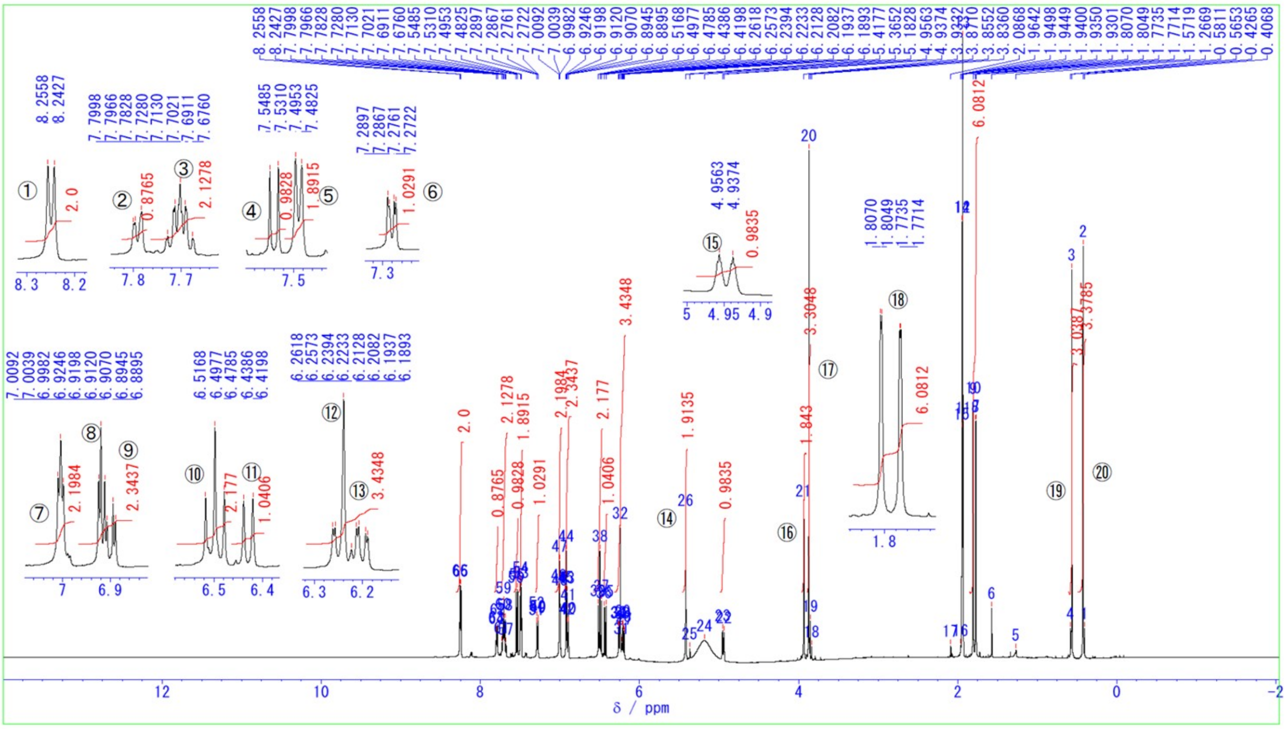

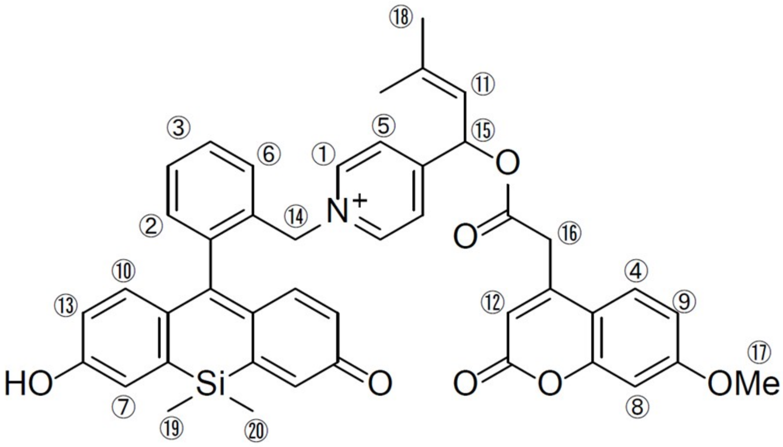

Compound 7

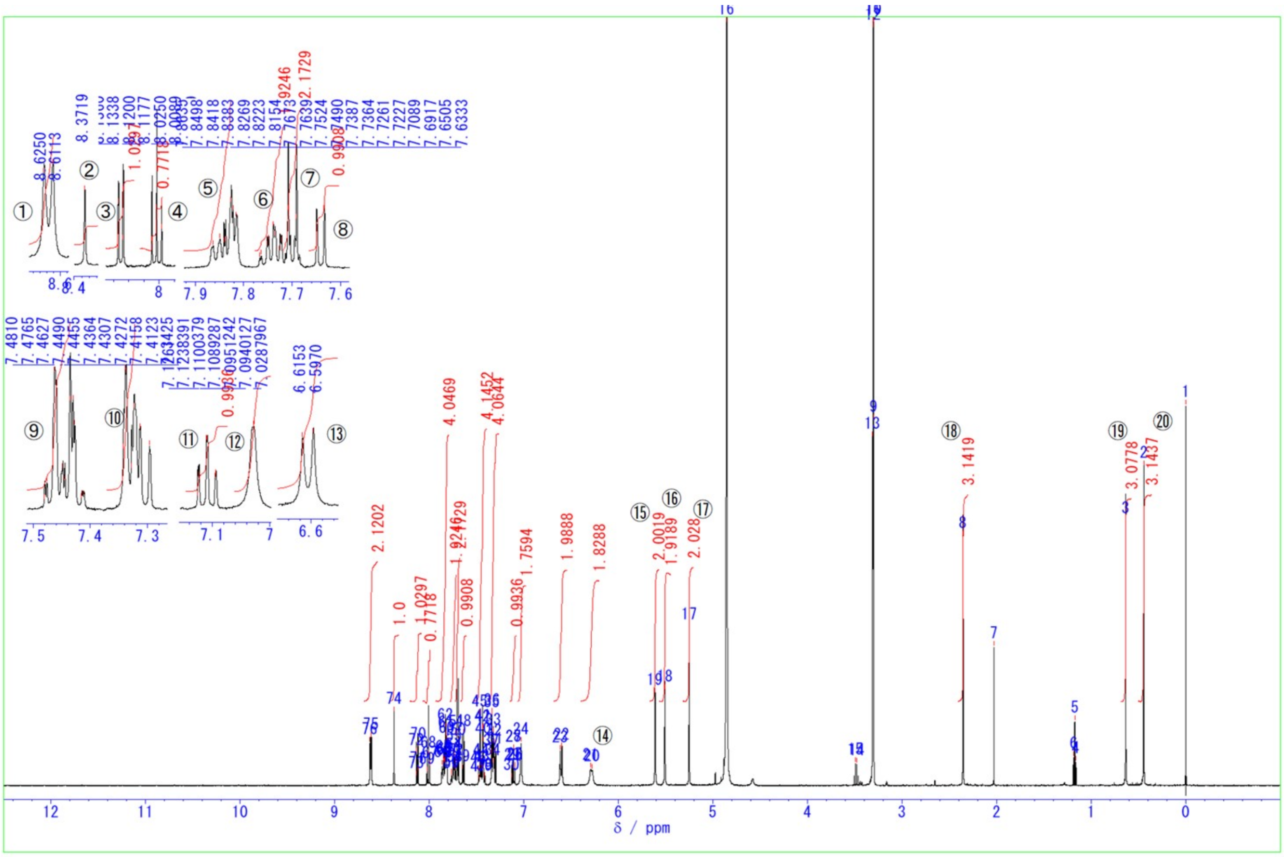

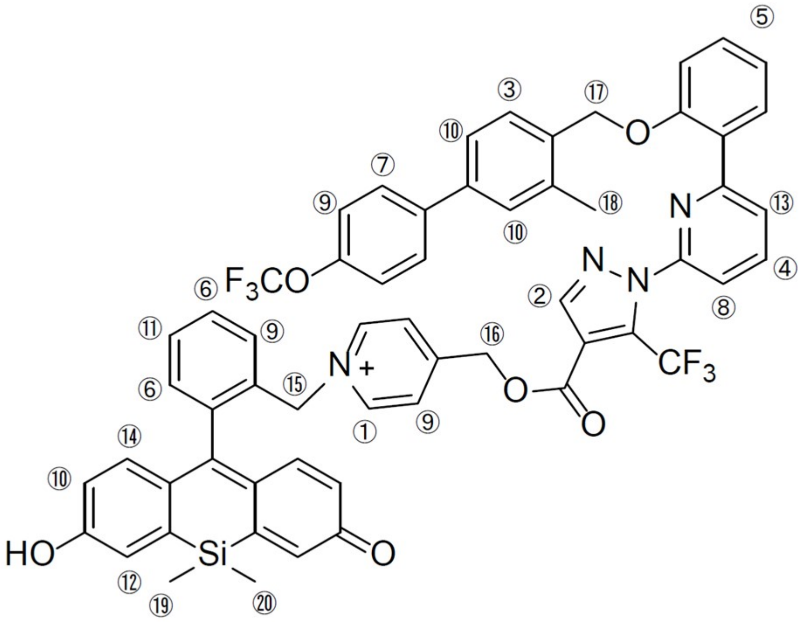

Supplementary Figures

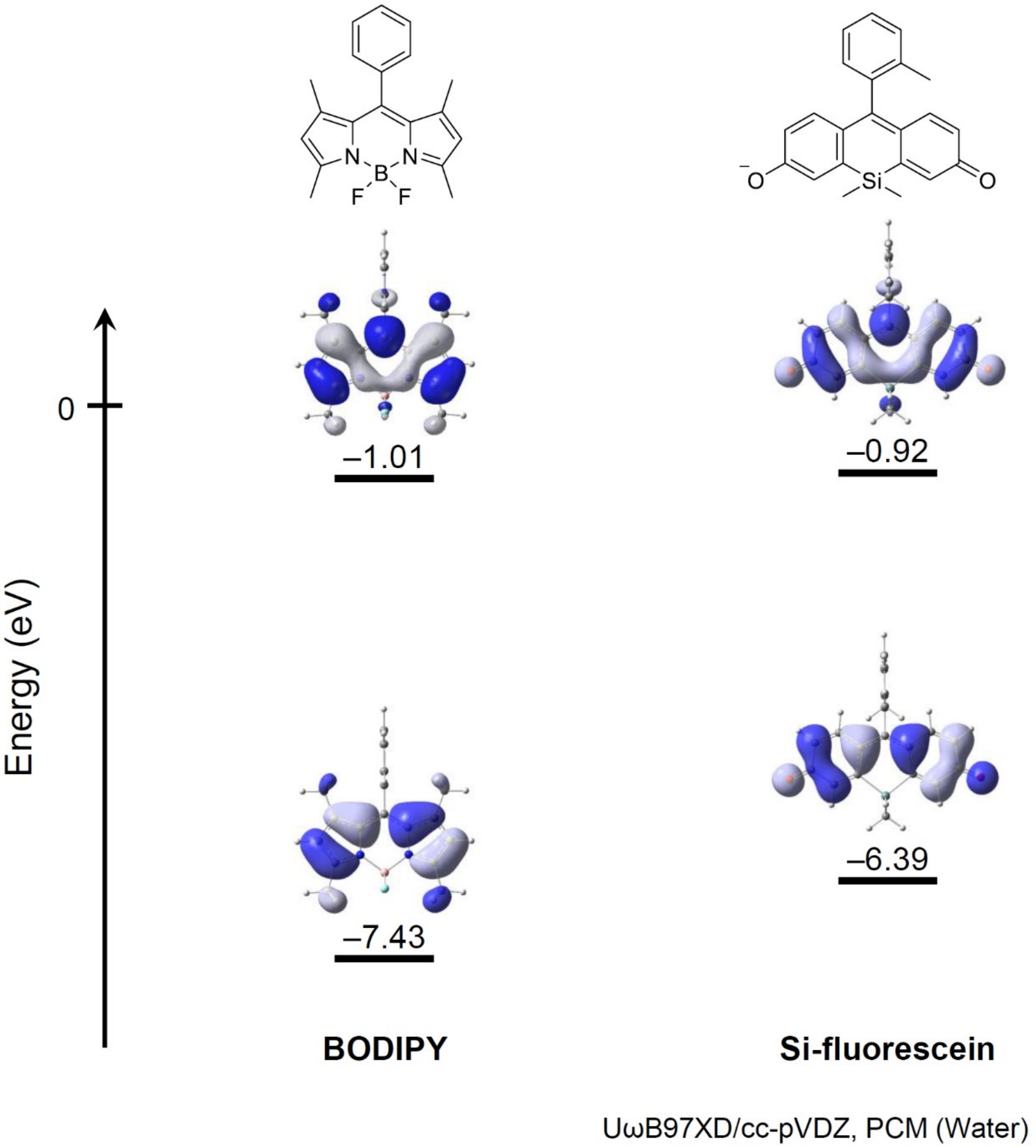

Fig. S1 Comparison of the frontier molecular orbitals of BODIPY and Si-fluorescein. Optimized structures and HOMO/LUMO isosurfaces with their corresponding energies (eV) calculated at the ωB97XD/cc-pVDZ level with PCM (water).

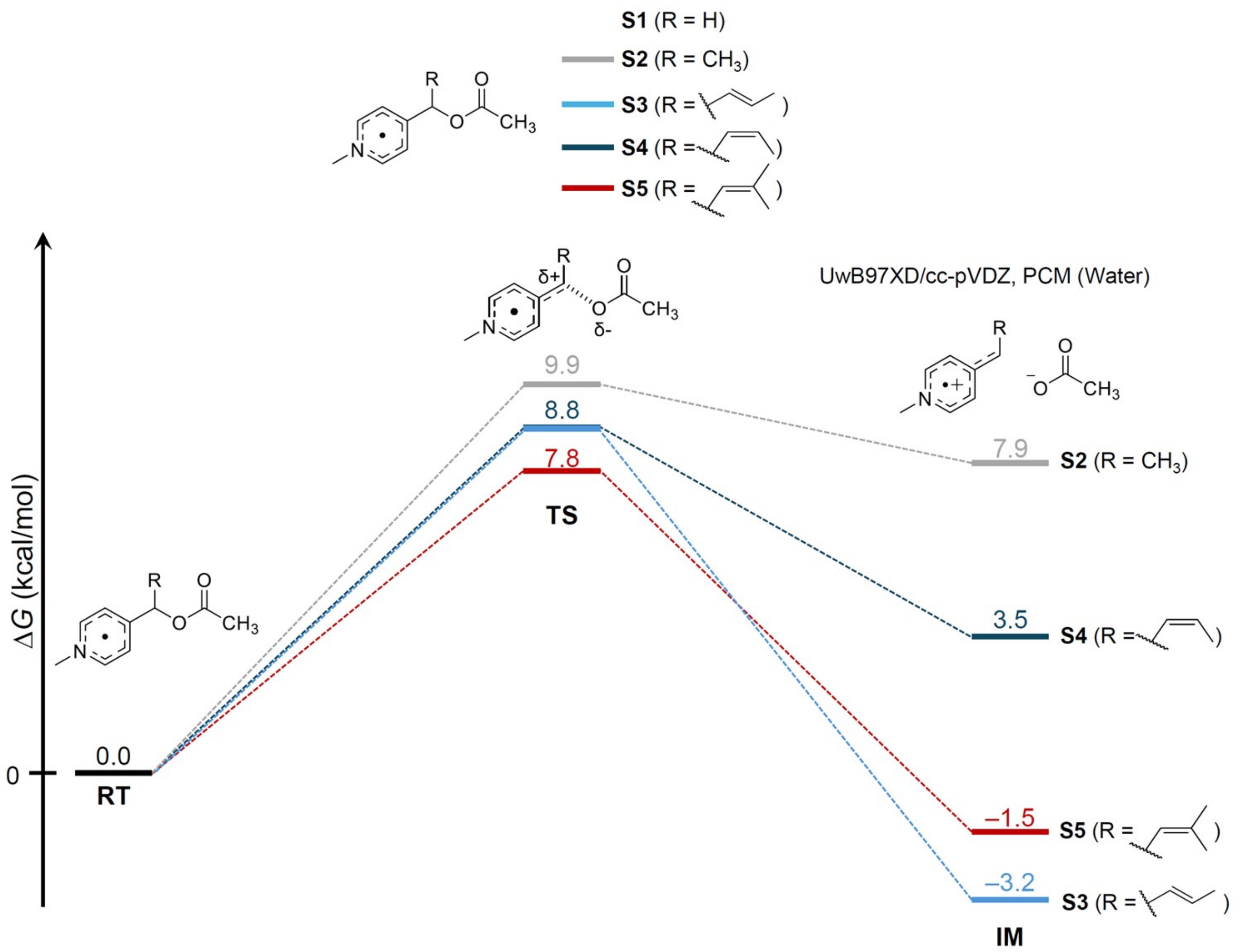

Fig. S2 Calculated energy diagram for the reaction pathway from the reactant (RT) to the intermediate (IM) via the transition state (TS). The activation energy (ΔE_a) and the overall Gibbs free energy change (ΔG) were obtained from quantum-chemical calculations performed at the UωB97X-D/cc-pVDZ level using the PCM solvation model (water).

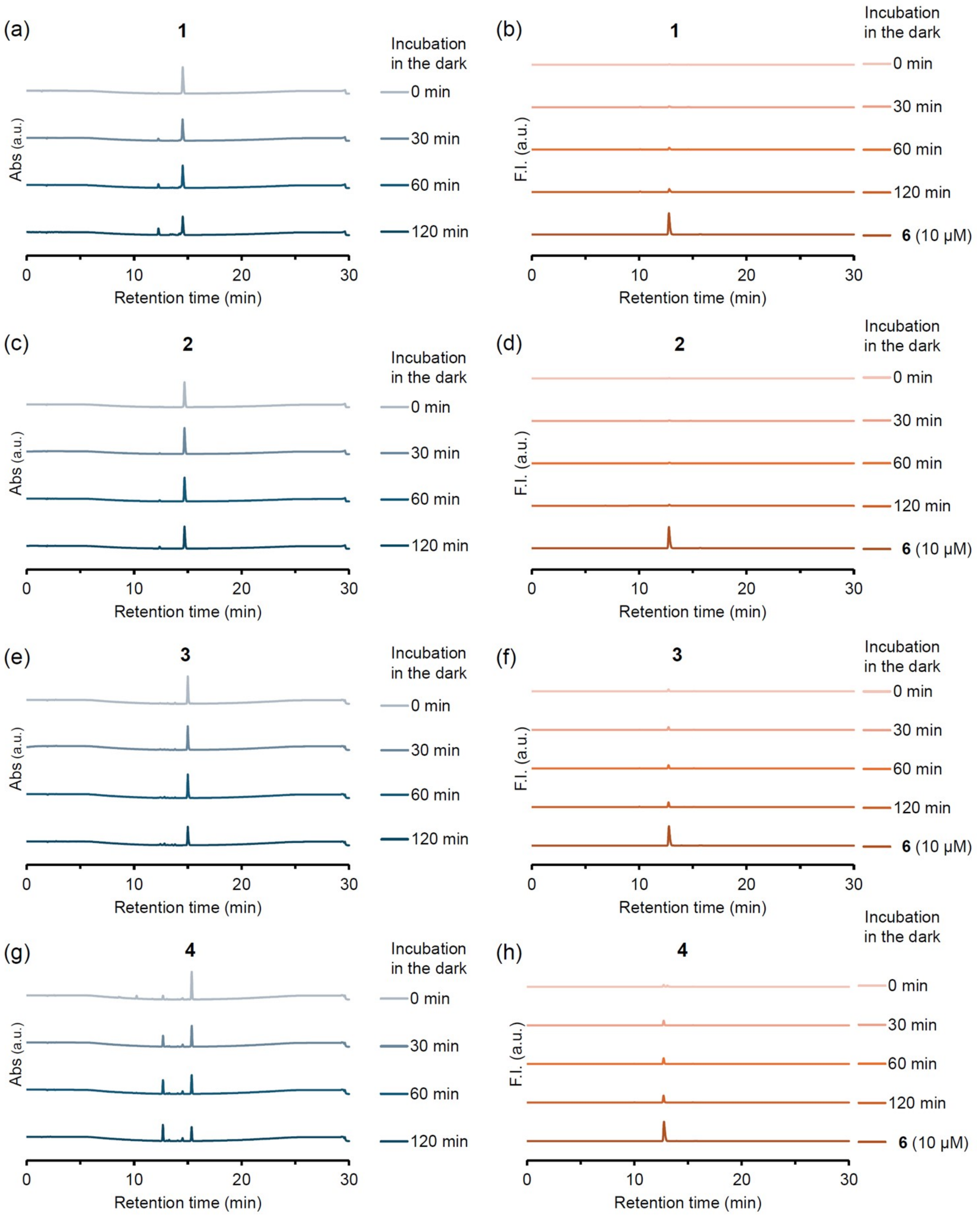

Fig. S3 Chromatograms of each compound (a, c, e, g) and **6** (b, d, f, h) after incubation in the dark for the indicated time. Samples were analyzed by HPLC after incubation in dark at 37 ºC in 100 mM HEPES buffer (pH 7.3, DMSO 0.1%).

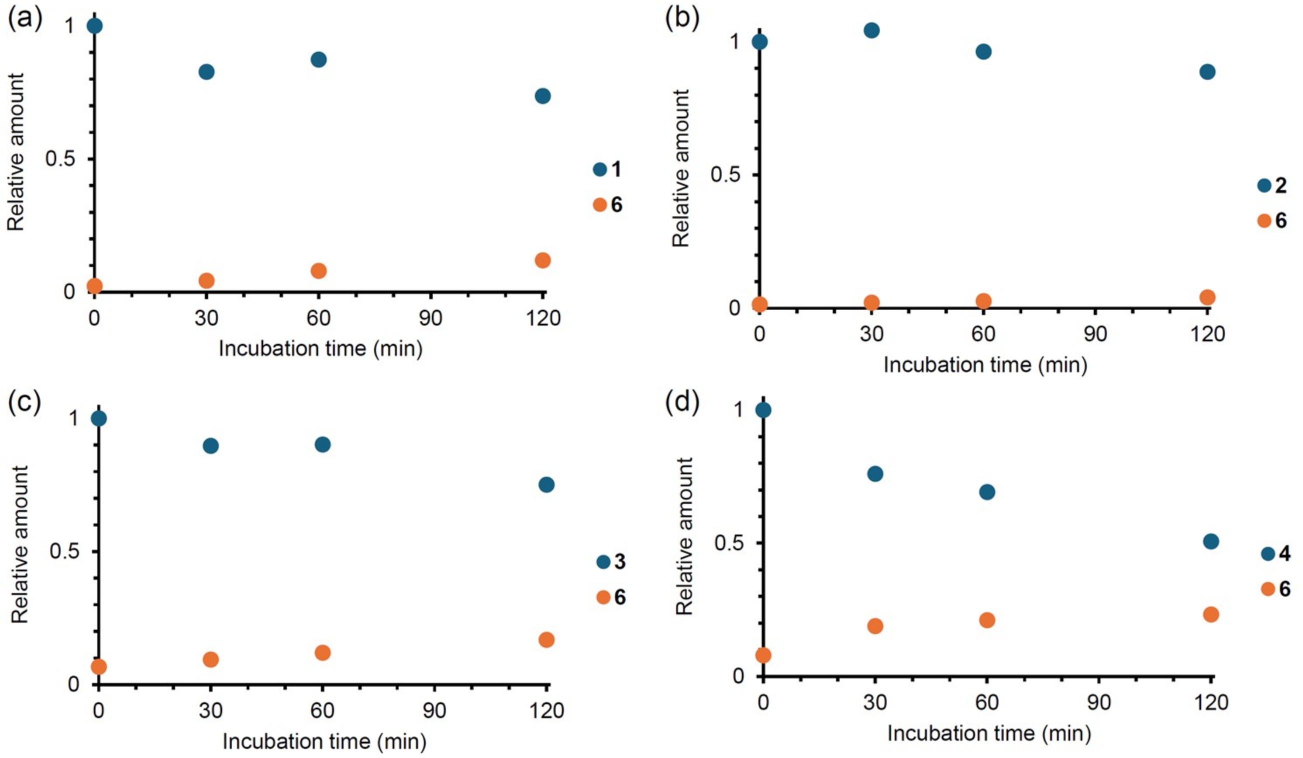

Fig. S4 Relative amount of the remaining amounts of compounds **1**–**4** and generation of **6** after incubation in the dark. Samples were analyzed by HPLC after incubation in dark for the indicated time at 37 ºC in 100 mM HEPES buffer (pH 7.3, DMSO 0.1%).

Table S1 Calculation of Φ_u of **1**

Actinometer (Reinecke's salt reaction)	1st	2nd	3rd	average	
A450 (before irrad.)	1.068E+00	1.055E+00	1.091E+00		(a)
[SCN⁻] (mol L^{-1})	2.484E-04	2.454E-04	2.538E-04		(b)
amount of SCN⁻ (mol)	1.246E-05	1.231E-05	1.273E-05		(c)
A'450 (after irrad.)	1.121E+00	1.117E+00	1.139E+00		(d)
[SCN⁻] (mol L^{-1})	2.607E-04	2.597E-04	2.649E-04		(e)
amount of SCN⁻ (mol)	1.307E-05	1.303E-05	1.328E-05		(f)
ΔSCN⁻ (mol)	6.149E-07	7.172E-07	5.550E-07		(g)
photons absorbed by RS (mol)	2.196E-06	2.561E-06	1.982E-06		(h)
Absorbance @600nm of RS	4.443E-01				(i)
$1\text{-}10^{-A}$	6.405E-01				(j)
photons irradiated to the cell (mol)	3.428E-06	3.999E-06	3.095E-06	3.507E-06	(k)
Absorbance @600nm of **1**	2.829E-01				(l)
$1\text{-}10^{-A}$	4.787E-01				(m)
photons absorbed by **1** (mol)	1.679E-06				(n)
photons absorbed by **1** (μmol)	1.679E+00				(o)
Uncaging reaction to release **6**					
Release of **6** from **1** (μmol L^{-1})	5.707E-01	5.972E-01	3.986E-01		(p)
Release of **6** from **1** (μmol)	1.712E-03	1.762E-03	1.176E-03		(q)
Φ_u	1.020E-03	1.049E-03	7.004E-04	9.231E-04	(r)
ε_{max} (598nm)	28424				(s)
$\Phi\varepsilon_{max}$	26.24				(t)

(a) Absorbance of Reinecke's salt solution at 450 nm before irradiation.
(b) Concentration of released SCN⁻ (mol L^{-1}) before irradiation: Calculated by dividing (a) by 4300, where 4300 is the molar extinction coefficient of $[Fe(SCN)]^{2+}$.
(c) Amount of SCN⁻ (mol) before irradiation: Calculated by multiplying (b) by the volume (0.00295 L) and the dilution factor (17).
(d) Absorbance of Reinecke's salt solution at 450 nm after irradiation.
(e) Concentration of released SCN⁻ (mol L^{-1}) after irradiation: Calculated by dividing (d) by 4300.
(f) Amount of SCN⁻ (mol) after irradiation: Calculated by multiplying (e) by the volume (0.00295 L) and the dilution factor (17).
(g) Change in SCN⁻ amount (delta SCN⁻ in mol): Calculated as (f) – (c), representing SCN⁻ generated by irradiation.
(h) Photons absorbed by Reinecke's salt (mol): Calculated by dividing (g) by 0.28 (the quantum yield of SCN⁻ release at 600 nm).
(i) Absorbance of the Reinecke's salt (RS) solution at 600 nm.

(j) Ratio of light absorbed by Reinecke's salt at 600 nm: Calculated as 1–10^(–(i)).

(k) Total photons irradiated to the cell (mol): Calculated by dividing (h) by (j).

(l) Absorbance of Compound **1** at 600 nm.

(m) Ratio of light absorbed by Compound 1 at 600 nm: Calculated as 1–10^(–(l)).

(n) Photons absorbed by **1** (mol): Calculated by multiplying (m) by the average value of (k).

(o) Photons absorbed by **1** (μmol): (n) converted from mol to micromoles.

(p) Concentration of 6 released from **1** (μmol L^{-1}).

(q) Amount of 6 released from **1** (μmol): Calculated by multiplying (p) by the sample volume (0.003 L).

(r) Quantum yield (Φ_u): Calculated by dividing (q) by (o).

Table S2 Calculation of Φ_u of **2**

Actinometer (Reinecke's salt reaction)	1st	2nd	3rd	average
A450 (before irrad.)	1.001E+00	1.049E+00	1.067E+00	
$[SCN^-]$ (M)	2.327E-04	2.438E-04	2.481E-04	
amount of SCN^- (mol)	1.167E-05	1.223E-05	1.244E-05	
A'450 (after irrad.)	1.036E+00	1.072E+00	1.089E+00	
$[SCN^-]$ (M)	2.408E-04	2.493E-04	2.533E-04	
amount of SCN^- (mol)	1.208E-05	1.250E-05	1.270E-05	
ΔSCN^- (mol)	4.090E-07	2.731E-07	2.593E-07	
photons absorbed by RS (mol)	1.461E-06	9.755E-07	9.260E-07	
Absorbance @600nm of RS	4.443E-01			
$1-10^{-A}$	6.405E-01			
photons irradiated to the cell (mol)	2.281E-06	1.523E-06	1.446E-06	1.750E-06
Absorbance @600nm of **2**	3.696E-01			
$1-10^{-A}$	5.731E-01			
photons absorbed by **2** (mol)	1.003E-06			
photons absorbed by **2** (μmol)	1.003E+00			
Uncaging reaction to release **6**				
Release of **6** from **2** (μmol/L)	1.439E+00	1.353E+00	9.325E-01	
Release of **6** from **2** (μmol)	4.317E-03	3.991E-03	2.751E-03	
Φ_u	4.305E-03	3.980E-03	2.743E-03	3.676E-03
ε_{max} (598nm)	37102			
$\Phi\varepsilon_{max}$	136.4			

Table S3 Calculation of Φ_u of **3**

Actinometer (Reinecke's salt reaction)	1st	2nd	3rd	average
A450 (before irrad.)	1.012E+00	1.027E+00	1.028E+00	
$[SCN^-]$ (M)	2.353E-04	2.388E-04	2.390E-04	
amount of SCN^- (mol)	1.180E-05	1.198E-05	1.199E-05	
A'450 (after irrad.)	1.014E+00	1.053E+00	1.033E+00	
$[SCN^-]$ (M)	2.359E-04	2.449E-04	2.403E-04	
amount of SCN^- (mol)	1.183E-05	1.228E-05	1.205E-05	
ΔSCN^- (mol)	3.074E-08	3.071E-07	6.700E-08	
photons absorbed by RS (mol)	1.098E-07	1.097E-06	2.393E-07	
Absorbance @600nm of RS	5.698E-01			
$1-10^{-A}$	7.307E-01			
photons irradiated to the cell (mol)	1.502E-07	1.501E-06	3.275E-07	6.595E-07
Absorbance @600nm of **3**	2.700E-01			
$1-10^{-A}$	4.629E-01			
photons absorbed by **3** (mol)	6.106E-08			
photons absorbed by **3** (μmol)	6.106E-02			
Uncaging reaction to release **6**				
Release of **6** from **3** (μmol/L)	1.111E+00	1.423E+00	1.563E+00	
Release of **6** from **3** (μmol)	3.334E-03	4.268E-03	4.690E-03	
Φ_u	5.461E-02	6.990E-02	7.681E-02	6.710E-02
ε_{max} (598nm)	27199			
$\Phi\varepsilon_{max}$	1825			

Cartesian Coordinates

S2$_{RT}$

Sum of electronic and thermal free energies

= -594.3276247 A.U

C	-4.54077614	0.41696734	0.36649271
H	-4.78644158	0.91280725	1.31467591
H	-5.00591772	-0.57803254	0.36698606
C	-2.25908028	1.26432004	0.75532196
C	-2.55907654	-0.68167148	-0.60054474
C	-0.92581995	1.27155069	0.47979960
H	-2.73200462	2.00506290	1.39943040
C	-1.22372304	-0.69859601	-0.88635685
H	-3.25943676	-1.41776396	-0.99260345
C	-0.33641925	0.28309533	-0.36959097
H	-0.31498188	2.05045453	0.93915735
H	-0.84900824	-1.49298168	-1.53609383
N	-3.10373169	0.28381788	0.24556037
C	1.12723059	0.25545709	-0.66681635
H	1.31440324	-0.54858297	-1.39132267
C	1.69370756	1.56676703	-1.19584394
H	1.19372184	1.83414015	-2.13791659
O	1.82992026	-0.06099543	0.58371271
C	2.93218803	-0.82142448	0.62892492
O	3.47589307	-0.97315196	1.70168664
H	-4.97034775	1.00644868	-0.46213089
C	3.43160126	-1.47430310	-0.63656571
H	4.36676537	-1.99463587	-0.40518572
H	2.69707529	-2.20401716	-1.00846848
H	3.60937944	-0.73939372	-1.43383806
H	1.53537585	2.38150249	-0.47404826
H	2.77420998	1.48539816	-1.38743735

S2$_{TS}$

Sum of electronic and thermal free energies

= -594.3117767 A.U

C	-4.87830761	-0.28428268	0.43701668
H	-5.13151949	0.50650160	1.15091352
H	-5.07336352	-1.25936628	0.89992686
C	-2.75985479	0.96237267	0.32903075
C	-2.84285177	-1.23019650	-0.53943961
C	-1.45080515	1.09162625	-0.03326606
H	-3.30719698	1.76047258	0.82806521
C	-1.53903095	-1.15747071	-0.92474190
H	-3.45637558	-2.11473204	-0.70414298
C	-0.76396734	0.02466501	-0.69589206
H	-0.94789346	2.03077760	0.19198273
H	-1.08842929	-2.02286431	-1.41130337
N	-3.46351885	-0.18293136	0.08892512
C	0.59465563	0.07751593	-1.05479083
H	0.93888598	-0.71590669	-1.71834116
C	1.39642611	1.33675097	-1.03150970
H	1.05088741	2.02326408	-1.82377665
O	1.49526689	-0.77447523	0.64026542
C	2.41732150	-1.66815630	0.59610128
O	2.91641249	-2.17663137	1.60928515
H	-5.49991609	-0.17526633	-0.46256363
C	2.90623132	-2.12137130	-0.77865941
H	3.75496538	-2.80841455	-0.67085914
H	2.09736827	-2.64140294	-1.31656524
H	3.21109710	-1.26176200	-1.39535681
H	1.30516295	1.85627926	-0.06757811
H	2.45846663	1.12376308	-1.20576664

S2$_{IM}$

Sum of electronic and thermal free energies

= -594.3149685 A.U

C	-4.43904456	0.13421756	0.61092058
H	-4.80936600	1.07957649	1.01894813

H	-4.47561630	-0.63941906	1.38885384
C	-2.37447120	1.45707045	0.43581896
C	-2.44277212	-0.68941150	-0.52932917
C	-1.07636797	1.62969206	0.04162152
H	-2.92125905	2.21991569	0.98748539
C	-1.15052459	-0.57488124	-0.94972363
H	-3.04516366	-1.57763407	-0.71381363
C	-0.39362385	0.60689353	-0.68253824
H	-0.58459911	2.56840519	0.29064046
H	-0.69893159	-1.40599198	-1.49102294
N	-3.05907471	0.31577473	0.15818861
C	0.94586415	0.70960975	-1.08644007
H	1.32219670	-0.08284440	-1.73278734
C	1.77643079	1.93364759	-0.92311213
H	1.45553424	2.71695243	-1.63285442
O	1.89892880	-0.34959311	0.77877115
C	2.79929854	-1.24700760	0.72006144
O	3.30454422	-1.81129756	1.70856042
H	-5.06655946	-0.16484455	-0.23733116
C	3.27978187	-1.65765639	-0.67833142
H	4.10762666	-2.37671521	-0.61522611
H	2.45140875	-2.11970514	-1.24006354
H	3.60818195	-0.77614955	-1.25172217
H	1.69364957	2.34502566	0.09277731
H	2.83294684	1.71270169	-1.11832593

S3$_{RT}$

Sum of electronic and thermal free energies

= -671.673631 A.U

C	-4.93484262	0.05303858	0.30334857
H	-5.20711496	0.57792675	1.22837943
H	-5.38747862	-0.94717868	0.32807535
C	-2.67392625	0.95254001	0.69580402
C	-2.91816764	-1.06638745	-0.56116556
C	-1.33190096	0.95446196	0.46213555
H	-3.17416046	1.72766800	1.27524277
C	-1.57643592	-1.08933175	-0.80607295
H	-3.60261473	-1.82475323	-0.93896363
C	-0.71148149	-0.07353821	-0.30929575
H	-0.73447334	1.76800621	0.87710086
H	-1.17639841	-1.91243354	-1.40246596
N	-3.49370847	-0.06600481	0.21821155
C	0.76916580	-0.13480365	-0.51545443
H	0.96487409	-0.83058841	-1.34586092
C	1.37161880	1.20603643	-0.84785361
C	2.19051611	1.91513680	-0.06866826
H	1.05771739	1.60556782	-1.81898998
O	1.36759769	-0.68361676	0.69956778
C	2.53058920	-1.35911729	0.68347206
O	2.97572335	-1.74224897	1.74272997
H	-5.35233575	0.60865074	-0.55414444
C	2.75170404	3.25925013	-0.42057241
H	3.85345016	3.23393539	-0.42732690
H	2.40644757	3.59959946	-1.40775758
H	2.45889981	4.01283037	0.32833790
C	3.20738091	-1.62347557	-0.63723247
H	4.17567024	-2.09434776	-0.43840025
H	2.59823563	-2.30349741	-1.25122235
H	3.35281758	-0.69280291	-1.20273819
H	2.48348825	1.50277875	0.90297885

S3$_{TS}$

Sum of electronic and thermal free energies

= -671.6596042 A.U

C	-4.93155513	-0.12945055	0.47795135
H	-5.11990676	0.61851976	1.25618745
H	-5.20858677	-1.11806351	0.86293729
C	-2.76228451	1.01320095	0.29094016
C	-2.93662573	-1.22691154	-0.43601579
C	-1.45016539	1.06410539	-0.07559425
H	-3.27796075	1.86682525	0.72832778
C	-1.63283748	-1.22804855	-0.82641838
H	-3.58318756	-2.09641445	-0.54676253
C	-0.80532335	-0.06897275	-0.66357609

H	-0.90888224	1.99669775	0.07382036
H	-1.21874836	-2.13905186	-1.25909005
N	-3.51422275	-0.12126009	0.13365261
C	0.56697041	-0.12198235	-1.02175674
H	0.81253876	-0.90801662	-1.73893385
C	1.41347708	1.07936156	-1.11511537
C	1.73328869	1.88469011	-0.09397766
H	1.82099632	1.28987409	-2.11160149
O	1.28627965	-1.10531622	0.52718364
C	2.41507314	-1.73740937	0.49322967
O	2.87871750	-2.30341057	1.48710833
H	-5.54520439	0.10226001	-0.40464597
C	2.58662231	3.10794815	-0.20521705
H	3.47977990	3.02115319	0.43469542
H	2.91463094	3.28339050	-1.24015397
H	2.03778106	3.99849393	0.14203376
C	3.17425173	-1.78210508	-0.82594336
H	4.13275612	-2.29696091	-0.68722349
H	2.58759692	-2.31941991	-1.58751795
H	3.35320247	-0.76528979	-1.20605458
H	1.37358179	1.62087791	0.90652348

$S3_{IM}$

Sum of electronic and thermal free energies

= -671.6786812 A.U

C	-4.68352487	-0.16083225	0.80684733
H	-5.18845514	0.80588458	0.88901287
H	-4.66893878	-0.65374002	1.78633664
C	-2.82033649	1.30253651	0.17153463
C	-2.51291767	-1.02152412	0.10555239
C	-1.52452822	1.51763651	-0.23365776
H	-3.51254451	2.12162373	0.35754413
C	-1.21568107	-0.87518054	-0.30876458
H	-2.97505772	-1.99562221	0.25865103
C	-0.65688833	0.42295841	-0.47021220
H	-1.21178935	2.54490980	-0.40376117
H	-0.58535834	-1.75218817	-0.51471049
N	-3.30453020	0.05450951	0.35269354
C	0.70489867	0.51720110	-0.90400345
H	1.11230132	-0.42994016	-1.27953492
C	1.57365524	1.63431157	-0.88325557
C	1.44712316	2.80512142	-0.18281132
H	2.50025899	1.50242990	-1.45367152
O	1.14501932	-2.56812600	-1.15823047
C	1.99493893	-2.91240740	-0.28009102
O	1.76731080	-3.58517091	0.74572398
H	-5.20961252	-0.79051999	0.07989295
C	2.44832194	3.90837538	-0.22183709
H	2.84418637	4.10701798	0.78788029
H	3.28784321	3.68026000	-0.89329009
H	1.97361460	4.84580953	-0.55666633
C	3.42704926	-2.40115868	-0.51299259
H	4.15741812	-2.92356639	0.12119457
H	3.71110786	-2.49930249	-1.57163090
H	3.46295137	-1.32541618	-0.26867339
H	0.59861206	2.95048961	0.49138250

$S4_{RT}$

Sum of electronic and thermal free energies

= -671.6713090 A.U

C	-4.90778571	0.13359987	0.15380309
H	-5.18518999	0.72234320	1.03812011
H	-5.34122976	-0.86985113	0.25713470
C	-2.66447092	1.11461359	0.42868740
C	-2.87232148	-1.02799941	-0.60645730
C	-1.32219498	1.11797333	0.19037592
H	-3.17862275	1.93938617	0.92047467
C	-1.53272580	-1.05071576	-0.85470034
H	-3.54263981	-1.83430975	-0.90162403
C	-0.68192969	0.02767262	-0.46725657
H	-0.74698249	1.98863676	0.50797507
H	-1.11863170	-1.92314829	-1.36489327
N	-3.46495687	0.03418724	0.06833850
C	0.79619336	-0.05675032	-0.67577986

H	0.97553825	-0.71986579	-1.53576651
C	1.45238621	1.27220490	-0.96384782
C	1.98077533	2.15251879	-0.10550855
C	2.06313629	2.07110293	1.39081915
H	1.50089036	1.22452325	1.80051380
H	3.11350986	1.97721018	1.71417995
H	1.68105153	3.00360481	1.83612619
H	1.46192636	1.52640338	-2.02854329
O	1.37270131	-0.71569098	0.50460098
C	2.57937844	-1.30651213	0.47165363
O	3.02542675	-1.74071121	1.51122659
H	-5.33934350	0.61486129	-0.74076476
C	3.30154728	-1.42377630	-0.84586975
H	4.27211585	-1.89785818	-0.66692581
H	2.72241453	-2.03942067	-1.54989374
H	3.44764979	-0.43565317	-1.30370520
H	2.40845395	3.06119075	-0.54456693

$S4_{TS}$

Sum of electronic and thermal free energies

= -671.6572309 A.U

C	-4.96723904	-0.13389536	0.43690555
H	-5.13550258	0.43548347	1.35917081
H	-5.30712792	-1.16398523	0.59007098
C	-2.80586659	1.00796171	0.26089817
C	-2.94365229	-1.25427361	-0.40528689
C	-1.48592170	1.05986016	-0.07339386
H	-3.34250190	1.86971613	0.65526513
C	-1.63032522	-1.25402118	-0.76438662
H	-3.57864787	-2.13330095	-0.50639078
C	-0.81673964	-0.08449322	-0.61004297
H	-0.95925187	2.00418576	0.05098607
H	-1.19753176	-2.17266584	-1.16056115
N	-3.54397504	-0.13915116	0.11898331
C	0.56318642	-0.12897558	-0.94668299
H	0.81345130	-0.87884210	-1.69994660
C	1.40594339	1.07981204	-0.99944395
C	1.73477836	1.90278533	0.00900251
C	1.36726981	1.75963310	1.45334317
H	0.83991646	0.81853236	1.64894888
H	2.27773035	1.78351444	2.07435732
H	0.74385377	2.60823229	1.78240666
H	1.81981208	1.29596495	-1.99047936
O	1.25915625	-1.22404832	0.50910903
C	2.40424427	-1.83011210	0.45329743
O	2.85519013	-2.45900772	1.41303214
H	-5.54436302	0.32020294	-0.38158367
C	3.19081599	-1.76044748	-0.84765971
H	4.14735485	-2.28412374	-0.73165330
H	2.62302616	-2.22996842	-1.66596433
H	3.37592360	-0.71461408	-1.13358237
H	2.36674945	2.76226490	-0.24074661

$S4_{IM}$

Sum of electronic and thermal free energies

= -671.6657107 A.U

C	-4.63113481	0.03765554	0.62625237
H	-4.75702647	0.66950115	1.51192133
H	-4.83406737	-1.00424218	0.89268779
C	-2.62154023	1.35109688	0.17824669
C	-2.61732679	-0.92826517	-0.38426618
C	-1.34550530	1.50312898	-0.29747541
H	-3.19127852	2.18001902	0.59490455
C	-1.34519555	-0.83474350	-0.87997611
H	-3.17625637	-1.86233849	-0.38000218
C	-0.64365714	0.39885381	-0.84593452
H	-0.89464742	2.49317953	-0.27243562
H	-0.85603391	-1.72821429	-1.26074456
N	-3.24989892	0.15034892	0.14595800
C	0.66548710	0.49524290	-1.38830784
H	0.95389763	-0.29126899	-2.08771057
C	1.58317685	1.58582813	-1.21981144
C	1.85465125	2.25727867	-0.07146385
C	1.34314348	1.92815894	1.29511475

H	0.92591631	0.91183904	1.32843617
H	2.16992286	1.99263558	2.01974065
H	0.57937334	2.65048201	1.63088861
H	2.19164451	1.82807096	-2.09801123
O	1.25354102	-1.42934532	0.42913877
C	2.41467226	-1.92894423	0.50583760
O	2.84282601	-2.65808497	1.42604407
H	-5.32261486	0.36004000	-0.16291298
C	3.37002013	-1.59118966	-0.65286859
H	4.34865823	-2.07582878	-0.52689821
H	2.92904795	-1.90979285	-1.61144839
H	3.50882450	-0.49983769	-0.71593842
H	2.58558875	3.07009612	-0.13594936

$S5_{RT}$

Sum of electronic and thermal free energies

= -710.9545991 A.U

C	-5.04135239	-0.05544771	0.18589825
H	-5.32546901	0.59846358	1.02118638
H	-5.45919495	-1.05351122	0.37141853
C	-2.81630262	0.99044239	0.33158317
C	-2.98757638	-1.25864323	-0.45313734
C	-1.47307118	0.98918255	0.08952991
H	-3.34479125	1.85774688	0.72495136
C	-1.64989705	-1.28560431	-0.70387098
H	-3.64401970	-2.10441846	-0.65488928
C	-0.81463021	-0.15513281	-0.43900279
H	-0.91433827	1.90180000	0.29990303
H	-1.22138500	-2.20141536	-1.11647222
N	-3.59765217	-0.14031454	0.10293751
C	0.66168766	-0.25228793	-0.66185988
H	0.82602956	-0.87029646	-1.55813386
C	1.36532875	1.06626020	-0.87162264
C	1.81908508	1.92872691	0.05049370
C	1.69480075	1.74589723	1.54033658
H	1.09859657	0.87003541	1.81602689
H	2.69456832	1.64159610	1.99506060
H	1.23960253	2.64523270	1.98719422
H	1.49706917	1.32837347	-1.92661079
O	1.21772003	-1.01125504	0.47346245
C	2.41313908	-1.61921806	0.40681325
O	2.83999226	-2.14221219	1.41355184
H	-5.48295766	0.34591971	-0.74261387
C	2.51381346	3.19761276	-0.36924429
H	3.52964963	3.24038007	0.05805339
H	2.59276351	3.28637419	-1.46162815
H	1.97399483	4.07884820	0.01545738
C	3.14987251	-1.64658736	-0.90769463
H	4.10877023	-2.15308611	-0.75623513
H	2.56775703	-2.19187077	-1.66526816
H	3.31972186	-0.62815675	-1.28345741

$S5_{TS}$

Sum of electronic and thermal free energies

= -710.9422563 A.U

C	-4.96240599	-0.13674060	0.44080461
H	-5.13341071	0.43793340	1.35945986
H	-5.30002182	-1.16666363	0.60075445
C	-2.80777921	1.01504285	0.24613143
C	-2.93146847	-1.25960864	-0.38353418
C	-1.48787762	1.06842647	-0.08728793
H	-3.35035046	1.88066510	0.62368513
C	-1.61784028	-1.25720815	-0.74070547
H	-3.56107510	-2.14384570	-0.47205668
C	-0.81077380	-0.08078881	-0.60303115
H	-0.96660465	2.01793872	0.01970259
H	-1.17862879	-2.17946439	-1.12109649
N	-3.53941257	-0.13964160	0.12349265
C	0.57067510	-0.12132365	-0.93982504
H	0.81902461	-0.86709761	-1.69811851
C	1.41328835	1.08471698	-0.99420774
C	1.74466539	1.91999414	0.00889783
C	1.33434762	1.72594836	1.44192944
H	0.81643963	0.77392214	1.59997735

H	2.22837848	1.74577609	2.08742415
H	0.69136316	2.55862366	1.77474086
H	1.83561724	1.28808740	-1.98500661
O	1.25790695	-1.24042484	0.49618730
C	2.40618212	-1.84030518	0.43981699
O	2.84269279	-2.50047495	1.38519414
H	-5.54133930	0.31030205	-0.38048890
C	2.60951042	3.12270768	-0.25221391
H	3.53973698	3.06546657	0.33738839
H	2.87407198	3.22287838	-1.31395393
H	2.09271669	4.04312024	0.06714846
C	3.21616556	-1.72168306	-0.84293633
H	4.17567383	-2.23965024	-0.72522210
H	2.66845593	-2.17038900	-1.68631917
H	3.39382796	-0.66521315	-1.09202650

$S5_{IM}$

Sum of electronic and thermal free energies
= -710.9569458 A.U

C	-4.62550452	-0.30141938	0.81769124
H	-5.07786907	0.68045628	0.98491457
H	-4.51932647	-0.82642445	1.77523053
C	-2.76140275	1.10738315	0.07416178
C	-2.60971759	-1.22008017	-0.20085274
C	-1.51488372	1.28085182	-0.47520133
H	-3.37107664	1.94339632	0.41153046
C	-1.36537462	-1.11249533	-0.76058387
H	-3.10201942	-2.17829712	-0.04144454
C	-0.76553426	0.16429656	-0.91692166
H	-1.13401127	2.29360634	-0.59679028
H	-0.77895505	-2.00223310	-1.01051507
N	-3.30225109	-0.12441341	0.20996861
C	0.50341558	0.28871695	-1.56021776
H	0.77812872	-0.53011735	-2.22931675
C	1.41765109	1.36832353	-1.43578862
C	1.70150809	2.09619749	-0.30773632
C	1.17822705	1.75470065	1.05809893
H	0.86873373	0.70135253	1.12886706
H	1.96796273	1.93746328	1.80379750
H	0.32973094	2.40101786	1.34076777
H	2.04277963	1.56149895	-2.31522182
O	0.90865990	-1.81175860	1.11251503
C	1.60865512	-2.13205271	0.11983032
O	1.21070732	-2.74072445	-0.91259077
H	-5.25962129	-0.88617322	0.14126824
C	2.67236260	3.23703548	-0.37265217
H	3.55627649	3.02728847	0.25279933
H	3.00509833	3.44799942	-1.39782744
H	2.20887379	4.14850253	0.04108995
C	3.08450307	-1.69867561	0.14190181
H	3.69652498	-2.29084176	-0.55352711
H	3.13918460	-0.64152618	-0.16997903
H	3.50071319	-1.76258110	1.15836189

S1 J. B. Grimm, T. A. Brown, A. N. Tkachuk, and L. D. Lavis, *ACS Cent. Sci.* **2017**, *3*, 975–985

S2 M. J. Frisch, G. W. Trucks, H. B. Schlegel, G. E. Scuseria, M. A. Robb, J. R. Cheeseman, G. Scalmani, V. Barone, G. A. Petersson, H. Nakatsuji, X. Li, M. Caricato, A. V. Marenich, J. Bloino, B. G. Janesko, R. Gomperts, B. Mennucci, H. P. Hratchian, J. V. Ortiz, A. F. Izmaylov, J. L. Sonnenberg, D. Williams-Young, F. Ding, F. Lipparini, F. Egidi, J. Goings, B. Peng, A. Petrone, T. Henderson, D. Ranasinghe, V. G. Zakrzewski, J. Gao, N. Rega, G. Zheng, W. Liang, M. Hada, M. Ehara, K. Toyota, R. Fukuda, J. Hasegawa, M. Ishida, T. Nakajima, Y. Honda, O. Kitao, H. Nakai, T. Vreven, K. Throssell, J. A. Montgomery Jr., J. E. Peralta, F. Ogliaro, M. J. Bearpark, J. J. Heyd, E. N. Brothers, K. N. Kudin, V. N. Staroverov, T. A. Keith, R. Kobayashi, J. Normand, K. Raghavachari, A. P. Rendell, J. C. Burant, S. S. Iyengar, J. Tomasi, M. Cossi, J. M. Millam, M. Klene, C. Adamo, R. Cammi, J. W. Ochterski, R. L. Martin, K. Morokuma, O. Farkas, J. B. Foresman, and D. J. Fox, *Gaussian 16*, Revision C.01, Gaussian, Inc., Wallingford CT, **2016**.

S3 (a) K. Fukui, *Acc. Chem. Res.* **1981**, *14*, 363. (b) K. Ishida, K. Morokuma, and A. Komornicki, *J. Chem. Phys.* **1977**, *66*, 2153. (c) C. Gonzalez and H. B. Schlegel, *J. Chem. Phys.* **1989**, *90*, 2154. (d) C. Gonzalez and H. B. Schlegel, *J. Phys. Chem.* **1990**, *94*, 5523.
